# Fast Confirmation of Antibody Identity by MALDI-TOF MS Fingerprints

**DOI:** 10.3390/antib9020008

**Published:** 2020-03-26

**Authors:** Georg Tscheuschner, Timm Schwaar, Michael G. Weller

**Affiliations:** Federal Institute for Materials Research and Testing (BAM), Division 1.5 Protein Analysis, Richard-Willstätter-Strasse 11, 12489 Berlin, Germany

**Keywords:** antibody ID, antibody registry, Research Resource Identifier, RRID, reproducibility, quality control, documentation, traceability, clones, biochemical reagents, diagnostics, immunoassays, ELISA, Western blot, immunohistochemistry, microarray, biosensor

## Abstract

Thousands of antibodies for diagnostic and other analytical purposes are on the market. However, it is often difficult to identify duplicates, reagent changes, and to assign the correct original publications to an antibody. This slows down scientific progress and might even be a cause of irreproducible research and a waste of resources. Recently, activities were started to suggest the sole use of recombinant antibodies in combination with the open communication of their sequence. In this case, such uncertainties should be eliminated. Unfortunately, this approach seems to be rather a long-term vision since the development and manufacturing of recombinant antibodies remain quite expensive in the foreseeable future. Nearly all commercial antibody suppliers also may be reluctant to publish the sequence of their antibodies, since they fear counterfeiting. De novo sequencing of antibodies is also not feasible today for a reagent user without access to the hybridoma clone. Nevertheless, it seems to be crucial for any scientist to have the opportunity to identify an antibody undoubtedly to guarantee the traceability of any research activity using antibodies from a third party as a tool. For this purpose, we developed a method for the identification of antibodies based on a MALDI-TOF MS fingerprint. To circumvent lengthy denaturation, reduction, alkylation, and enzymatic digestion steps, the fragmentation was performed with a simple formic acid hydrolysis step. Eighty-nine unknown monoclonal antibodies were used for this study to examine the feasibility of this approach. Although the molecular assignment of peaks was rarely possible, antibodies could be easily recognized in a blinded test, simply from their mass-spectral fingerprint. A general protocol is given, which could be used without any optimization to generate fingerprints for a database. We want to propose that, in most scientific projects relying critically on antibody reagents, such a fingerprint should be established to prove and document the identity of the used antibodies, as well as to assign a specific reagent to a datasheet of a commercial supplier, public database record, or antibody ID.

## 1. Introduction

Antibodies still belong to the most important biochemical reagents today. Billions of Euros are spent every year [1], which means that the production and sale of antibodies for diagnostic and other analytical purposes is a significant economic factor. On the other side, antibodies are suspected [2] to be a frequent reason for the lack of reproducibility in biomedicine, biochemistry, and bioanalysis [3,4,5,6]. The wrong application of antibodies might cause substantial financial and reputational damage in research and other fields. Quite a few efforts have been performed to resolve or at least reduce these problems. Recently, some simple rules have been proposed to improve the validation level of antibody-based techniques [7]. One of the most basic rules, which seems to be violated in more than 50% of all publications [8], is the need for the precise and unambiguous identification of a reagent. Besides a lack of awareness under many scientists, the suboptimal information policy of some companies might be a factor. This lack of transparency is often justified with intellectual property, which in the end may make an experimental protocol useless and the respective paper irreproducible. Some antibody identification databases, e.g., in [9,10] (https://scicrunch.org/resources/; https://antibodyregistry.org/; https://www.citeab.com/), have been established, which seem to gain more and more popularity. However, there is nearly no way to verify such information, and hence any mistakes, such as duplicates in database entries or mislabeled/changed reagents, cannot be recognized easily. Thus, there seems to be an urgent need for an independent confirmatory verification path for complex biochemical reagents, such as antibodies.

There is an active group in the antibody community suggesting abandoning the use of polyclonal and non-recombinant monoclonal antibodies due to a lack of “validation”, in favor of the use of entirely recombinant reagents in combination with their sequence information [11,12,13,14]. This approach seems to be quite elegant to get rid of many problems at once. However, when this approach is assessed in more detail, some practical limitations emerge [15,16,17]. First of all, a sequence of a protein is only a small fraction of the analytical validation [7,18] since the amino acid sequence does not reveal much information about the (analytical) properties of a protein. Nevertheless, the primary structure has valuable information—mainly an unambiguous label, such as a unique antibody ID. Unfortunately, the determination of the sequence of an antibody is a relatively expensive and lengthy matter. It may be feasible if the cell clone and hence the DNA or RNA are accessible. If the cell line or DNA is not available, the sequencing on the protein level is quite a costly and error-prone endeavor. Sometimes, the hope is expressed that commercial suppliers of antibodies might include sequence information in their datasheets in the future. We think that this is not very likely since this would make the “copying” of an antibody too easy. Patent protection seems to be an unfeasible option, either because of the lack of an inventive step, or the prohibitive costs and efforts. Therefore, we concluded that a fast and easy method to identify an antibody without access to the sequence would be highly desirable.

It is evident that mass spectrometry is one of the most powerful methods for protein analysis today. Unfortunately, most protocols are quite complex and may need several days of highly qualified work to be completed. Only a smaller number of specialized laboratories can offer this service, also at a considerable cost, which is not attractive for most routine applications. MALDI-TOF MS is one of the mass spectrometric methods that demands only limited sample preparation and does not require a chromatographic step. Therefore, MALDI-TOF MS is quite popular for a quick analysis of relatively complex samples, delivering a result often in only some minutes. The difficulty in obtaining quantitative results with MALDI-TOF MS is not relevant for identification tasks, which are pursued in the application presented.

Up to now, (peptide) mass fingerprinting has mainly used for microbial identification [19,20,21,22,23], e.g., for pathogenic *Legionella* [24] or for the analysis of peptide mixtures obtained after tryptic digestion of 2D-gel electrophoretically separated proteins [25,26,27,28,29,30,31].

**Figure 1:**
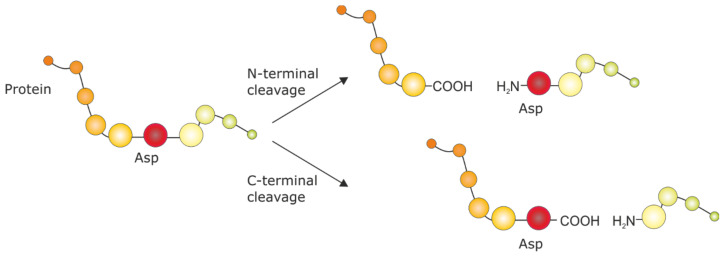
Chemical fragmentation by formic acid cleavage at the N-terminal or C-terminal Asp peptide bond [32].

Here we propose a simplified method to identify a monoclonal or recombinant antibody solely based on the mass spectrometric fingerprints of its peptide fragments obtained by heating with a weak acid [32,33,34,35,36,37,38] (Figure 1). The method is based on the comparison of the mass spectra with a library entry but does not require any sequence information or access to the respective cell line.

First of all, a short purification and enrichment step on magnetic protein G [39,40] particles may be performed, optionally. This cleanup removes any unwanted matrix compounds, such as salts, as well as stabilizers, such as albumin. In addition, an estimation of the enriched IgG amount can be performed with simple protein determination methods if the IgG concentration is unknown. In the case of a pure antibody preparation, this cleanup step is unnecessary.

The elution of the IgG from the protein G particles is performed with 2% formic acid, which is the cleavage reagent of the protein, as well in the next step. Formic acid cleavage of proteins is a proven method for the generation of relatively defined peptides. A simple scheme is shown in Figure 1. The main advantage of this approach is the parallel denaturation of essentially all IgG since the cleavage is performed at 90 °C (or higher) [41,42]. TCEP should be added, which significantly improved the formation of significant fragments by the reduction of disulfide bonds. However, this required the execution of an additional purification step on reversed-phase tips to get rid of the TCEP and its oxidation products. The elution of the peptides is performed directly in the tips with the MALDI matrix (CHCA) solution, which is then directly applied to a target plate. The MALDI spectra are performed in the linear mode in a range of 1000–10,000 *m*/*z*. In our case, we restricted the calibration with poly(methyl methacrylate) polymers (PMMA) to a range between 1000 and 7500 *m*/*z*.

Finally, the peaks of the mass spectrum are matched with reference spectra stored in a database. A Java software tool was used for this purpose. A set of 89 monoclonal antibodies of unknown specificity was used as a test system. It could be shown that the respective clones could be easily identified from the in-house database, even if the identity was not known to the operator (blinded experiment).

## 2. Materials and Methods

For optimization of the method parameters, the BAM-mab 02 (E1) antibody (2.0 mg/mL) distributed by BAM, Berlin, and produced by InVivo BioTech, Hennigsdorf, was used. The validation was performed using the antibodies BAM-mab 01 (CBZ) (1 mg/mL) and Anti-FLAG^®^ M2 (1.0 mg/mL), among others. All antibodies were stored at 4 °C in PBS solution.

### 2.1. Clean-Up and Enrichment of IgG

A custom-made magnetic rack was used to hold eight LoBind Eppendorf tubes (2 mL). The particle slurry was carefully mixed by vortexing before dispensing 20 µL of the suspension into an Eppendorf tube. A washing step was performed by adding 500 µL of binding buffer (PBS, pH 7.4) and the particles were resuspended by vortexing. The washing liquid was removed while the magnetic particles were held back in the magnetic rack. This washing step was repeated once before adding 495 µL of binding buffer and 5 µL of the antibody stock solution of 2 mg/mL for a total incubation volume of 500 µL. The particles were resuspended by careful vortexing, and the tube was placed on an “Eppendorf Thermo Mixer C” at 24 °C and 950 rpm for one hour. Afterwards, the liquid was removed in the magnetic rack, and the particles were washed twice with 500 µL of purified water. A total of 150 µL of 2% formic acid (FA) was added to the particles, and the tube was placed on the mixer at 24 °C and 950 rpm for two minutes. The elution fraction was collected in a 0.2 mL PCR tube.

### 2.2. Protein Cleavage

A total of 10 µL of 50 mM aqueous tris(2-carboxyethyl) phosphine hydrochloride (TCEP) was added to the 150 µL 2% FA solution containing the purified antibody. The PCR tubes were then placed on the Eppendorf Thermo Mixer with a SmartBlock PCR 96 at 90 °C and 800 rpm for 5 h. Dithiothreitol (DTT) was also tested as a reducing agent during protein cleavage. In that case, a 50 mM DTT solution was added to the 150 µL of 2% FA solution containing the purified antibody. In addition, cleavage times of one, two, three, four, and sixteen hours were tested.

### 2.3. Microscale Solid-Phase Extraction

Samples were allowed to cool to room temperature after protein cleavage. For the removal of TCEP and for peptide enrichment, Thermo Scientific Pierce C18 Tips (10 µL) were used. The tips were wetted and equilibrated by aspirating and discarding 10 µL of acetonitrile (ACN) followed by 0.1% of trifluoroacetic acid (TFA) in purified water. This was repeated once before dispensing and aspirating 10 µL of the sample for 20 times. Washing was performed by aspiration of 10 µL of 0.1% TFA in purified water for five times. Peptides were eluted with 2 µL of an α-cyano-4-hydroxycinnamic acid (CHCA) solution (2:1 ACN:H_2_O, 0.1% TFA) and the mixture was directly deposited on a target spot on the MALDI plate.

### 2.4. MALDI-TOF-MS Calibration

All measurements were performed using a Bruker Autoflex II Smartbeam™ in linear mode. A mixture of two PMMA polymers with different peak molecular weights (Mp = 1960 and Mp = 5050) was used as a calibrant. The compounds were dissolved in tetrahydrofuran (THF) to form a 3 mg/mL solution. A total of 20 µL of this solution was mixed with 50 µL of a 20 mg/mL trans-2-(3-(4-tert-butylphenyl)-2-methyl-2-propenylidene)malononitrile (DCTB) solution in THF. A total of 0.5 µL of the resulting mixture was deposited next to each sample spot. A calibration file (see Table S1, Supplementary Materials) with reference masses for the n-mers of PMMA was then used to (re)calibrate the instrument before measuring each sample.

### 2.5. General Protocol

After optimization, all subsequent antibody samples were treated using one protocol: Clean-up and enrichment of IgG were performed as described above in Section 2.1. For each antibody sample, an amount of about 10 µg was used. Afterwards, three replicates of 10 µL aliquots were used from the elution fraction to determine the antibody concentration via a Bradford Assay (see Supplementary Materials for the detailed protocol).

Protein cleavage was carried out by adding 10 µL of 50 mM TCEP to the remaining 120 µL of 2% FA solution containing the purified antibody and placing the PCR tubes on an Eppendorf Thermo Mixer C with a SmartBlock PCR 96 at 90 °C and 800 rpm for 5 h (see Section 2.2). Subsequently, microscale solid-phase extraction was used to purify the peptides, as described in Section 2.3, and the calibration was performed as described in Section 2.4 with a mixture of PMMA polymer for MALDI MS measurements. The obtained spectra were examined by an in-house Java software tool, ABID (see Supplementary Materials).

## 3. Results

### 3.1. Optimized Method Parameters

**Figure 2:**
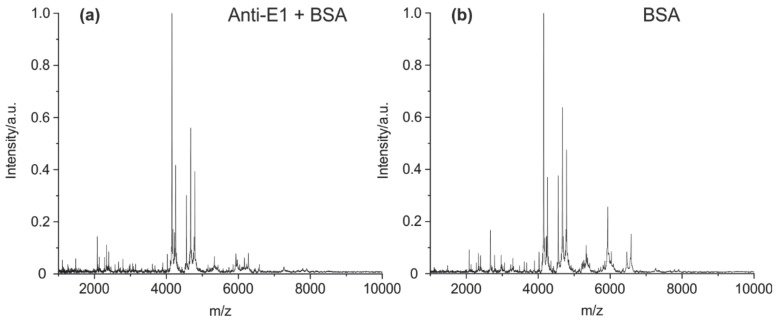
MALDI-TOF MS spectra of (**a**) mouse IgG + BSA (concentration ratio 1:10) and (**b**) cleaved bovine serum albumin (BSA, 100 µg/mL). The dominance of the BSA peaks may impede the analysis of the antibody fragments. Therefore, the separation of IgG from any additives by magnetic particles coated with protein A or G is recommended, if the purity and/or concentration of the IgG sample is unknown.

The optimization of the cleavage protocol and detection by MALDI-TOF MS was performed using an in-house-developed mouse antibody against estrone (E1) [43]. The antibody cleavage (10 µg of IgG2) was performed with 2% formic acid. It has been shown that even 1 µg may be enough to get comparable results in terms of peptide abundance and signal-to-noise ratios. Protein-G-based immunoprecipitation with magnetic beads was applied to remove any salts and other stabilizing agents (such as BSA). Without the removal of BSA, almost all peptide peaks in the mass spectrum belong to BSA (Figure 2a,b).

**Figure 3:**
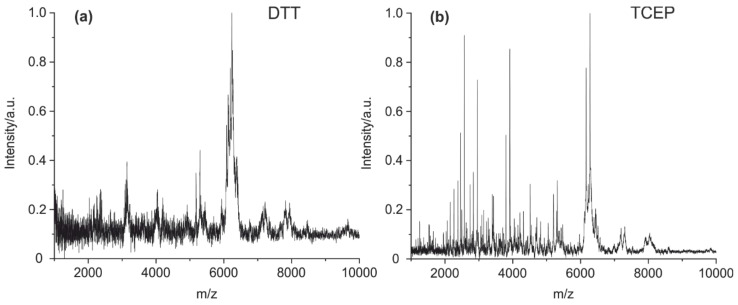
MALDI-TOF MS spectra of (**a**) mouse IgG1 cleaved with DTT as a reductant and (**b**) mouse IgG1 with TCEP as an additive. It is evident that TCEP seems to be the preferred reagent for the cleavage of disulfide bridges.

Furthermore, two different reducing agents (DTT and TCEP) were tested as a reductant in the cleavage solution containing 2% of formic acid. Antibodies contain many intra- and intermolecular disulfide bridges, which often prevent the generation of a sufficient set of characteristic peptide fragments. The intended cleavage of the disulfide bridges using DTT yielded a spectrum with only a few peaks (Figure 3a). The reduction with TCEP results in a spectrum with a high number of clearly resolved peaks (Figure 3b). It has to be noted that in contrast to TCEP, DTT is known to be a poor reduction agent at an acidic pH [44].

**Figure 4:**
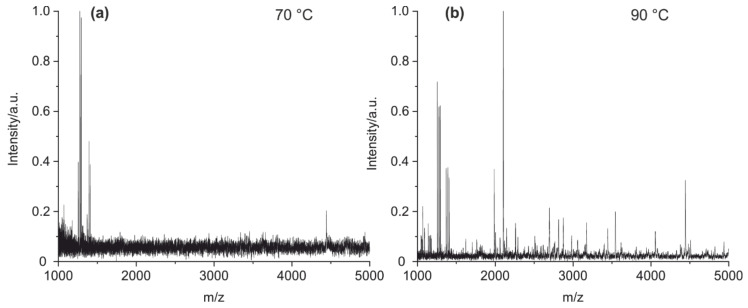
MALDI-TOF MS spectra of (**a**) mouse IgG1 cleaved at 70 °C and (**b**) mouse IgG1 cleaved at 90 °C. The difference is obvious. It can be suspected that some antibodies are not sufficiently denatured at 70 °C.

In a further step, the cleavage temperature was optimized (Figure 4). It could be shown that at least 90 °C is needed to hydrolyze all tested antibodies with sufficient efficiency. Higher temperatures have been avoided for practical reasons.

**Figure 5:**
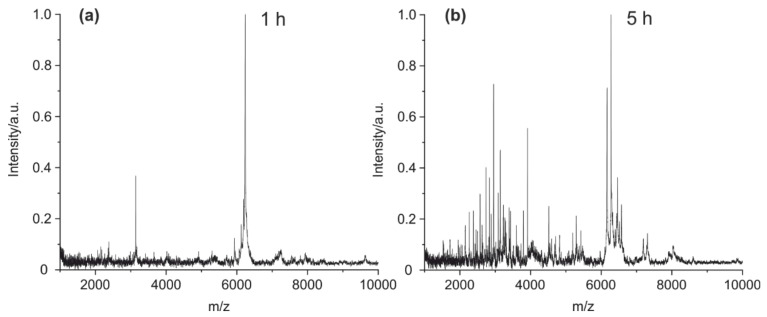
MALDI-TOF MS spectra of (**a**) mouse IgG1 cleaved at 90 °C for one hour and (**b**) mouse IgG1 cleaved for five hours.

For the optimization of the cleavage time, the antibody was treated with 2% FA for one, two, three, four, five, and sixteen hours (results in Supplementary Materials Figure S1) at 90 °C. After one hour (Figure 5a), only a few peptides peaks could be observed in the mass spectrum. The number of peaks increased with time, and the highest yield of peptide peaks was reached after five hours (Figure 5b).

**Figure 6:**
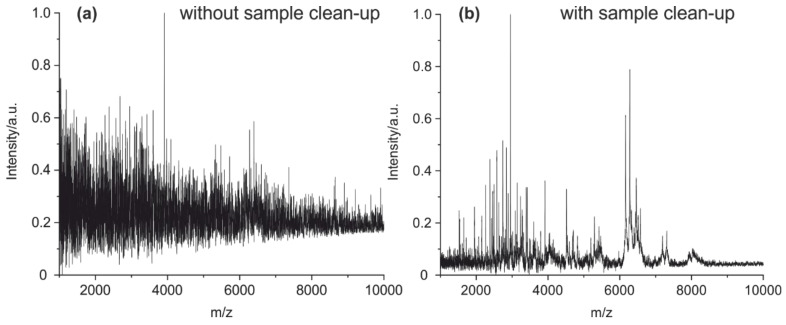
MALDI-TOF MS spectra of a cleavage solution of a mouse IgG1 (**a**) without any treatment and enrichment and (**b**) with clean-up and enrichment on a C18 pipet tip absorber.

In addition, the necessity of a sample clean-up using solid-phase extraction (SPE) on a pipette tip was examined. This step removes TCEP and its oxidation products, which could interfere with the MALDI MS measurements. In addition, the peptides get concentrated. Without the clean-up by SPE, no fingerprint spectrum could be obtained (Figure 6).

**Figure 7:**
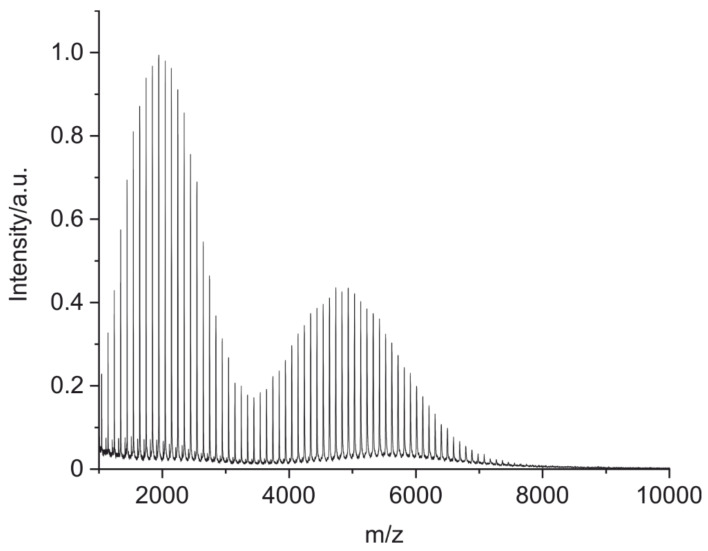
A MALDI-TOF MS spectrum of a calibration polymer poly(methyl methacrylate) (PMMA) with an *m*/*z* range of 1000–7500. The polymer was dissolved in tetrahydrofuran (THF) (c = 3 mg/mL) and mixed with the matrix trans-2-(3-(4-tert-butylphenyl)-2-methyl-2-propenylidene)malononitrile (DCTB, c = 20 mg/mL in THF) in a 2:5 (V:V) ratio. A total of 0.5 µL of the mixture was deposited on the MALDI plate.

Calibration of the MALDI-TOF mass spectrometer was performed using a mixture based on a poly(methyl methacrylate) (PMMA) polymer with two different molar masses in their peak maxima (M_p_ = 1960 g/mol and M_p_ = 5050 g/mol) (Figure 7). Using this method, it was possible to calibrate the instrument up to 7500 *m/z*.

**Figure 8:**
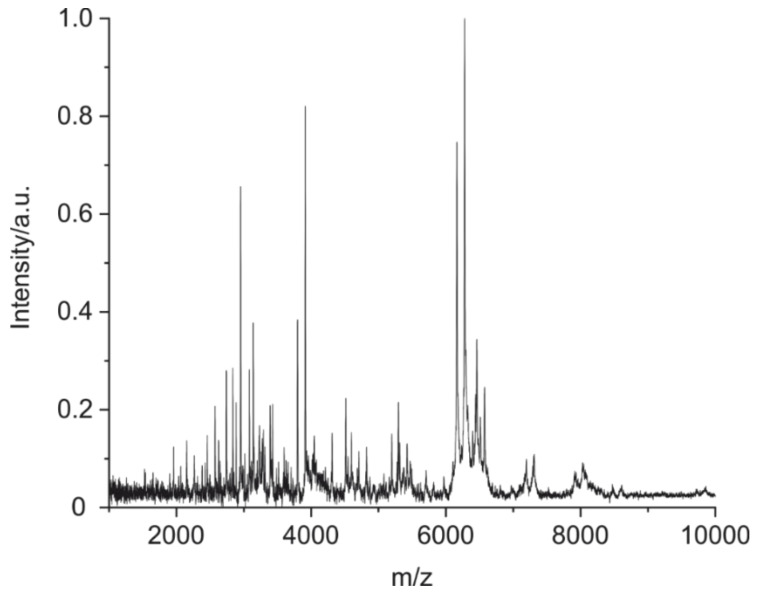
MALDI-TOF MS spectra of a cleaved mouse IgG1 (BAM-mab 02 (E1)) under optimized conditions: 1) Optional enrichment of IgG by magnetic particles with immobilized protein G; 2) cleavage of 1 µg of IgG with 2% of formic acid plus TCEP at 90 °C for 5 h; 3) cleanup on pipet tip SPE; 4) elution with alpha-cyano-4-hydroxycinnamic acid in acetonitrile/water/trifluoroacetic acid (TFA) and application on MALDI target (a calibration polymer was applied on a separate spot on the target); and 5) the MALDI-TOF MS measurement was performed in the linear, positive mode.

In Figure 8, a typical MALDI-TOF MS spectrum of a mouse IgG1 is shown, obtained by application of the optimized purification, cleavage, and measurement conditions. The information-rich spectrum seems to be well-suited for the intended identification/confirmation purpose.

### 3.2. Fingerprints of Known Monoclonal Antibodies (IgG1 and IgG2)

**Figure 9:**
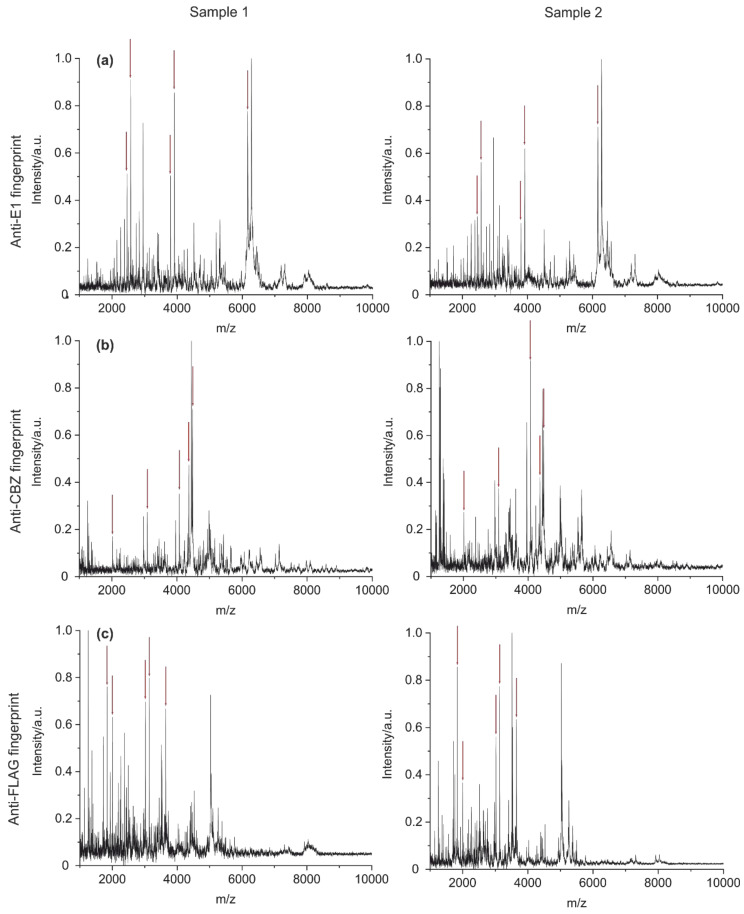
Comparison of fingerprints between three monoclonal antibodies E1 (**a**), CBZ (**b**), and FLAG M2 (**c**). Red arrows mark the five most intense signals from peptides that only occur in the fingerprint spectrum of the respective antibody. Multiple measurements from individual samples were taken. The unique peaks for each antibody were also found in the replicate.

After optimization of the method with the E1 antibody (BAM-mab 02 (E1)), the procedure was tested with two additional monoclonal antibodies known from the literature [43,45]. Peptide mass fingerprints of an anti-carbamazepine antibody (clone BAM-mab 01 (CBZ)) and an anti-FLAG antibody (clone M2) were made. A comparison of the three spectra is shown in Figure 9. Red arrows highlight the five most intense signals from peptides that only occur in the fingerprint spectrum of the respective antibody. These unique peaks were also well reproducible, as shown in the measurements of a replicate.

### 3.3. Validation with a Set of 89 Clones

In order to validate the presented method, about a hundred randomly selected monoclonal antibodies were used to examine the uniqueness of the antibody fingerprint. It was tested whether each antibody gave a different fingerprint in a reproducible manner and whether it would be possible to distinguish each of them. Nearly all of the hundred antibodies originated from mice and only one was of human origin. They were delivered in varying concentrations ranging from 0.06 mg/mL to 4.5 mg/mL. No information was given regarding their antigenic specificity, affinity, constitution, nor amino acid sequence. For about 80% of the antibodies, a provisional class or subclass information was given, with some being more specific than others; for example, “mouse IgG” for one antibody and “mouse IgG2b κ” for another antibody.

For some of the hundred antibodies, the concentration after elution from Protein G was significantly lower than indicated (<1 µg, see Supplementary Materials Figure S1). For some of these samples, it was not possible to acquire satisfactory spectra. However, antibodies with a concentration above 1 µg always gave decent fingerprint spectra. The fingerprints of all 100 antibodies used in this study are shown in the Supplementary Materials.

#### 3.3.1. Software

**Figure 10:**
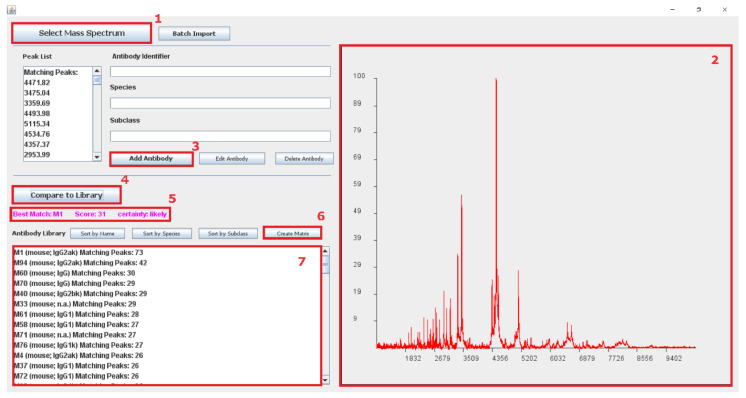
Screenshot of the software tool “ABID” for creating databases for antibody fingerprints and searching the database for matches. (**1**) “Select Mass Spectrum” allows the user to upload an antibody fingerprint spectrum (as ASCII file) into the software. A peak picking algorithm is automatically detecting the peaks in the spectrum, creating a list mass of antibody peptide peaks. The uploaded spectrum and detected peaks are shown in Window (**2**). The user can create a new entry to the database by filling in an antibody identifier, species, and antibody subclass and subsequently adding it by pressing (**3**): “Add Antibody”. The spectrum can be compared to all library entries by pressing (**4**): “Compare to Library”. The best match is shown in Window (**5**) with a given certainty based on a calculated score. Button (**6**), “Create Matrix”, creates a correlation matrix, as shown in Figure 5, correlating all peptide mass lists from the library with each other. All library entries are sorted by the number of matching peaks (± value of predefined resolution) with the uploaded spectrum and shown in Window (**7**).

For handling large amounts of antibody spectra, the Java software tool “ABID” was programmed. A screenshot of the software is shown in Figure 10. This Java tool allows the creation of antibody fingerprint libraries. A new entry in the library is created by uploading a MALDI spectrum of a cleaved antibody into the software. The software is automatically identifying peaks in the spectrum, creating a mass list from these detected peaks. Adding several antibody fingerprints creates a library that can be used to identify antibodies from a given spectrum.

To assign a new antibody sample to a library record, a fragment spectrum can be uploaded, peaks will be identified, and the peak list will be compared to all library entries. A peak from the uploaded spectrum is identified as “Matching Peak” (see Figure 10, Window 5) when the *m/z* from a library entry is equal or in range of the predefined resolution (here: 2 Da). The library list is sorted by the “Matching Peaks”, calculated and presented in Figure 10, Window 7. A more detailed guide on how to use the software tool can be found in the Supplementary Materials (Figure S3).

An additional feature of the software tool is the function to create a correlation matrix. To generate this matrix, all the antibody fingerprints from the library are matched with each other. The resulting matching peaks are divided by the total number of peaks in the mass list. This results in values between 0 and 1, where 0 means no matched peaks and 1 that all the peaks are matching.

#### 3.3.2. The Specificity of Antibody Fingerprints

**Figure 11:**
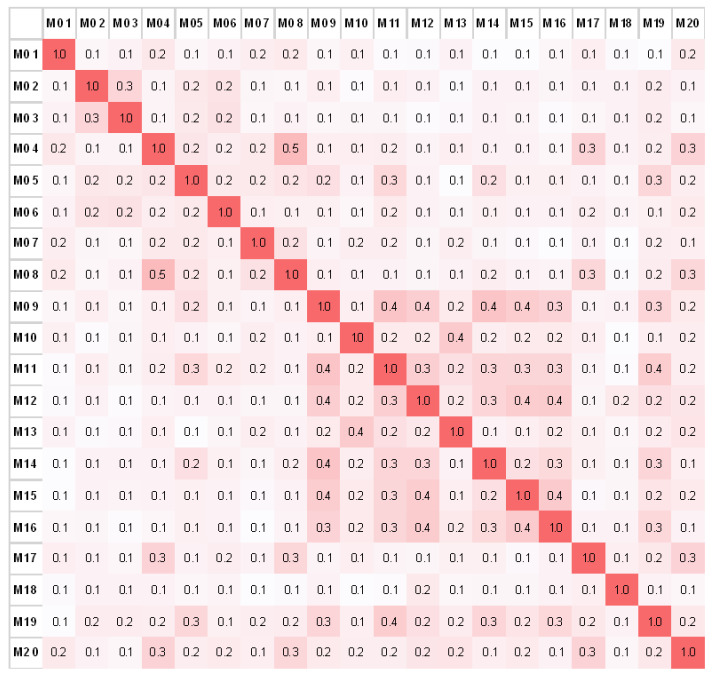
The correlation matrix with matching peak percentage plotted over a selection (M01–M20) of antibody fingerprints. The higher the color intensity, the higher the spectral overlap between two antibody fingerprints.

Using the software tool described above, a correlation matrix was produced (Figure 11). For the full-sized matrix with all antibodies used in this study see Figure S4 (Supplementary Materials). The matrix shows the relative match for each antibody compared to all the other antibodies. The number of matching peaks for every antibody is given in a normalized value. The intensity in color correlates with a rising number of matching peaks. In general, the average correlation between all 89 antibody spectra is 21% ± 10%. The lowest correlation was found for the single human antibody (M18) with a 12% ± 2.6% spectral overlap with any other (murine) antibody.

#### 3.3.3. Blinded Random Sampling

To test the reproducibility of the presented method, some blinded random samples were selected. Five samples of the above mentioned 89 antibodies were chosen randomly and reexamined with the workflow described. Subsequently, the five newly acquired spectra from the selected antibodies were matched against all 89 library entries. Table 1 shows the results of the random sampling. The screenshots of the software tool determining the best matches for each blinded random sample can be found in Figure S5 (Supplementary Materials). All five antibody samples were successfully matched to their corresponding library entries. A score was calculated as the difference of the number of matching peaks between the best and second-best match for each fingerprint spectrum.

## 4. Discussion

### 4.1. Method Optimization

Prior to protein cleavage, a sample clean-up has to be considered. With high concentrations of albumin (BSA) present, almost no antibody peaks could be detected. Accordingly, a simple subtraction of the BSA peaks from the spectrum usually does not give a satisfactory antibody fingerprint. Most antibody peptides seem to be suppressed [46] by BSA peptides during the MALDI MS measurements (Figure 2a).

In addition, the reduction of all disulfides is critical to the efficient cleavage of the antibody. Using a low pH during protein cleavage, TCEP was reported to be significantly more effective than DTT [44]. This is in good agreement with the results we found in this study. The performance of DTT was poor, whereas TCEP gave an excellent fingerprint spectrum (Figure 3).

Furthermore, the temperature during cleavage is a crucial factor since incomplete denaturation of the antibody can result in a dramatic drop in peak number (Figure 4). The influence of temperature on the unfolding of antibodies has been studied before [47]. We have chosen 90 °C for the method presented as a temperature at which every domain of the protein should be completely unfolded 

In order to identify a given protein by peptide mass fingerprinting, mass accuracy and peptide peak abundance during MS measurements need to be sufficiently high. The presented method uses the linear mode of MALDI-TOF MS. Since measurements in linear mode are usually much more sensitive than in reflector mode, more peptide peaks are detected. However, the lack of ion focusing increases time-of-flight spread during mass analysis, so that adequate mass calibration becomes a more critical factor. For this reason, the polymer PMMA [48,49,50] was chosen as a novel calibrant in this context (Figure 7). The broad mass range and the high number of peaks of the PMMA mixture make the calibration feasible without the need for expensive and unstable peptides as standards. Furthermore, a higher calibration range is needed since peptide fragments generated by formic acid cleavage are longer compared to the standard cleavage by tryptic digestion. This method of calibration would also work reasonably well using the reflector mode. For the application of openly accessible antibody fingerprint databases, a standardized calibration should be used, and the authors highly recommend using PMMA as a calibrant for this purpose.

Even though most of the experiments were performed using 10 µg of antibody in this study, it could be shown that down to 1 µg good spectra can be obtained (Figure 8). This can be useful for expensive antibodies or samples of low concentration. When creating an antibody fingerprint for a library entry, the authors recommend using 10 µg of protein. Lower concentrations may be used in a library comparison.

### 4.2. Validation of Antibody Identification

As shown in the correlation matrix (Figure 11 and Figure S4), some antibodies seem to have little to no spectral overlap with other antibodies, for example, M18 and M94. This implicates a “uniqueness” of the antibody fingerprint. M18 is the only human antibody among 88 mouse antibodies in the library and should have no structural similarities with any other antibody in the library. The M94 antibody, on the other hand, originated from mouse, but has the subclass IgG2a κ, with only five antibodies having the same subclass. Since IgG1 is the most abundant subclass in the library, fingerprints of antibodies with this subclass tend to have a higher overlap than others. For instance, this is the case for M15 and M16 (40% overlap).

The results of the random sampling in Table 1 show that the method presented is able to identify randomly picked antibodies out of a sum of 89 different antibodies present in a given library. A score was introduced, which is an indication of the confidence of a matched antibody. These scores differed from sample to sample, with the lowest being 15 and the highest being 41.

### 4.3. Advantages/Applications

The presented method, which is new and easy to use, has several potential applications. It enables the user to identify (or confirm) an antibody among several other antibodies that have, for example, the same antigen in a fast and straightforward way. Furthermore, using this method, it is easier to avoid buying the same antibody twice that has been given another name or arbitrary product number if the supplier or a customer uploaded the pattern of a clone. In the same way, it is possible for antibody users to identify wrongly labeled antibodies. Future applications may also involve species and subclass characterization of the antibodies as an alternative to ELISA or lateral flow assays (LFA). But more work on this subject is needed to explore this potential feature fully. Lastly, for the purpose of experiment reproducibility, antibodies of unknown sequence should be published together with their specific mass fingerprint. This spectrum could be included in the supplementary information, additionally uploaded to open data repositories, and be used by other groups in order to identify the antibody used in the study. Finally, it would be very advantageous to combine an RRID or other antibody ID with this peptide mass fingerprint to confirm the identity of the antibody independently.

### 4.4. Limitations

In this study, it could be shown that the method presented is able to distinguish 89 different monoclonal antibodies. However, with a rising number of antibodies in a library, the chance of a false positive match increases. Nevertheless, in most cases, only a few relevant clones (against the same or similar antigen) need to be compared. Distinguishing even a small number of antibodies can be challenging when they are too similar. For example, point mutations in the amino acid sequence could probably not be recognized by this method. Likewise, the identification of daughter clones or batch-to-batch quality control is not recommended with this method as long it is not combined with other criteria, such as the precise determination of the antibody molecular mass [51,52,53,54] and charge heterogeneity [55].

## 5. Conclusions

Quality control of antibody-based methods is a challenging task. Many reproducibility issues in biochemical and medical studies are attributed to shortcomings in this context [3]. The repeated sequencing of any antibody reagent would be the most desirable approach [14]. Unfortunately, due to severe limitations in the aspects cost, time, and intellectual property, it does not seem very likely that this approach would be a realistic solution in the short term. Therefore, we developed a quick and easy fingerprint method based on MALDI-TOF MS to compare and identify monoclonal antibodies of hybridoma or recombinant source. We propose to attach such an antibody fingerprint to any scientific publication or other studies critically relying on antibodies, irrespective of in-house or commercial origin. In projects for hybridoma or other antibody development, it could be a component of good laboratory practice (GLP), in order to characterize all positive clones [56] also with their respective peptide fingerprints. In the future, the determination of the species and the antibody subclasses might also be included, which would provide an extra benefit of the technique. The combination with antibody IDs, such as RRIDs [9], would be a powerful way to improve the traceability of all antibody reagents. Finally, such data should be made available in the public domain in the sense of an Open Data [57] approach.

## Figures and Tables

**Table 1:** The number of matching peaks identified by the software for the best match of the randomly selected antibodies M1, M10, M36, M79, and M82. The score gives the difference between the number of matching peaks of the best and second-best match.

Random Sample	Best Match (# Matching Peaks)	Score (∆ Matching Peaks)
**M01**	**M01** (73)	31
**M10**	**M10** (97)	35
**M36**	**M36** (107)	41
**M79**	**M79** (89)	27
**M82**	**M82** (72)	15

## Supplementary Materials

The following are available online at https://www.mdpi.com/2073-4468/9/2/8/s1: Table S1: Reference mass list for the potassium adducts of the PMMA mixture; Figure S1: Optimization of protein cleavage time; Figure S2: Antibody amount of each sample prior to protein cleavage determined via Bradford assay; Figure S3: Screenshot of the software tool ABID; Figure S4: Correlation matrix of all 89 antibodies; Figure S5: Software-based results of the blinded samples; Fingerprint spectra of all antibodies; Software_and_Spectra.zip.
