# Supplementary material for: Fast Confirmation of Antibody Identity by MALDI-TOF MS Fingerprints
Source: Antibodies (Basel). 2020 Mar 26;9(2):8. doi: 10.3390/antib9020008 (PMC7362173; doi:10.3390/antib9020008)
Supplement: Supplementary file 1 [file antibodies-09-00008-s001.zip › antibodies-09-00008-s001.pdf]

antibodies

Supplementary Materials

of the paper

Fast Confirmation of Antibody Identity by MALDI-TOF-MS Fingerprints

Georg Tscheuschner#, Timm Schwaar# and Michael G. Weller [ORCID]*

Federal Institute for Materials Research and Testing (BAM), Division 1.5 Protein Analysis, Richard-Willstätter-Strasse 11, 12489 Berlin, Germany

# Both authors contributed equally to the paper.

* Correspondence: michael.weller@bam.de; Tel.: +49-30-8104-1150

Materials

BAM-mab 01 (Anti-CBZ) and BAM-mab 02 (Anti-E1) monoclonal antibodies were obtained from BAM Division 1.8. One hundred randomized monoclonal antibody samples were purchased from InVivo BioTech GmbH (Hennigsdorf, Germany). Bovine serum albumin (BSA, 23209), Coomassie Bradford reagent (23200), trifluoroacetic acid > 99,5 % (TFA, 85183) and Pierce C_{18} Tips, 10 µL bed (87782) were purchased from Thermo Fisher Scientific (Waltham, Massachusetts, USA). α-Cyano-4-hydroxycinnamic acid > 99 % (CHCA, 70990), trans-2-[3-(4-tert-butylphenyl)-2-methyl-2-propenyl-idene] malononitrile > 99 % (DCTB, 87884) and dithiothreitol > 99 % (DTT, D0632) were purchased from Sigma-Aldrich (St. Louis, Missouri, USA). Protein G Mag Sepharose Xtra (11545664) was purchased from GE Healthcare (Chicago, Illinois, USA). Formic acid 98 % (FA, 94318) was obtained from Honeywell Fluka. Tris(2-carboxyethyl)phosphine hydrochloride 98 % (TCEP, 580560) was purchased from Calbiochem (San Diego, USA). Acetonitrile 99.95 % (ACN, 2697) and tetrahydrofuran 99.9 % (THF, 775) were purchased from Th. Geyer GmbH & Co. KG (Renningen, Germany). Lab water was obtained from a Milli-Q water purification system (Millipore, Bedford, MA, USA) with a resistivity of > 18.2 Ω and TOC value of < 5 ppb. Poly(methyl methacrylate) (PMMA) was obtained from PSS Polymer Standard Service GmbH.

MALDI TOF MS calibration

For MALDI MS calibration, a mixture of two poly(methyl methacrylate) polymers (PMMA) with average molar masses of 1960 g/mol and 5050 g/mol were used. In order to enhance ionization and facilitate peak recognition, a few crystals of potassium trifluoroacetate were added to the trans-2-[3-(4-tert-butylphenyl)-2-methyl-2-propenyliden]malononitrile (DCTB) MALDI matrix before mixing it with the polymer. A reference list containing the masses of the potassium adducts of the n-mers of the polymer mixture was then used to calibrate the instrument. **Table S1** contains this information.

Table S1. Reference mass list for the potassium adducts of the PMMA mixture.

PMMA n-mer + K^+	m/z, average mass
10-mer	1042.27
15-mer	1542.85
20-mer	2043.43
25-mer	2544.01
30-mer	3044.59
35-mer	3545.17
40-mer	4045.75
45-mer	4546.33
50-mer	5046.91
55-mer	5547.48
60-mer	6048.06
65-mer	6548.64
70-mer	7049.22
75-mer	7549.80

Optimization of protein cleavage time

Optimization of protein cleavage time was performed by applying different reaction times to the same antibody sample during protein cleavage. **Figure S1** shows the fingerprint obtained from antibody cleavage at one hour (a), two hours (b), three hours (c), four hours (d), five hours (e), and sixteen hours (f). The most information-rich spectrum was obtained after 5 h of protein cleavage.

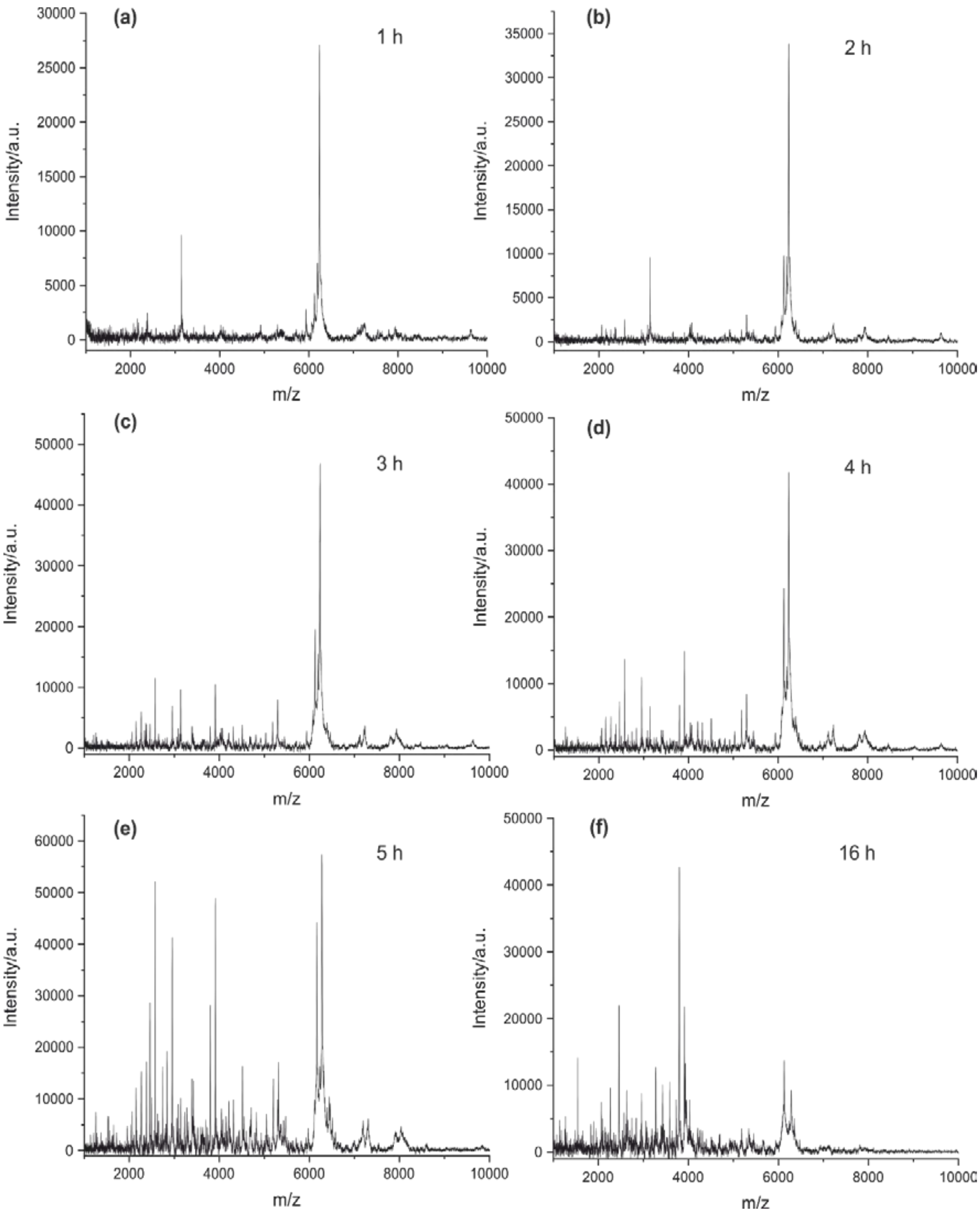

Figure S1. Optimization of protein cleavage time. MALDI-TOF-MS spectra of mouse IgG_1 cleaved at 90 °C for one (a), two (b), three (c), four (d), five (e), and sixteen hours (f).

Bradford assay

Prior to protein cleavage, the concentration of the antibodies was determined via Bradford Assay. For each sample, three aliquots of 10 µL were mixed with 200 µL of Bradford reagent on a microtiter plate. The calibration of the assay was performed with the human therapeutic antibody Adalimumab. The mixture of sample aliquots and Bradford reagent was put in the dark for 10 min before measuring the absorption of each cavity with a microplate reader (595 nm) The concentrations were automatically calculated using the software *Gen5*. **Figure S2** shows the antibody amount of each sample prior to protein cleavage.

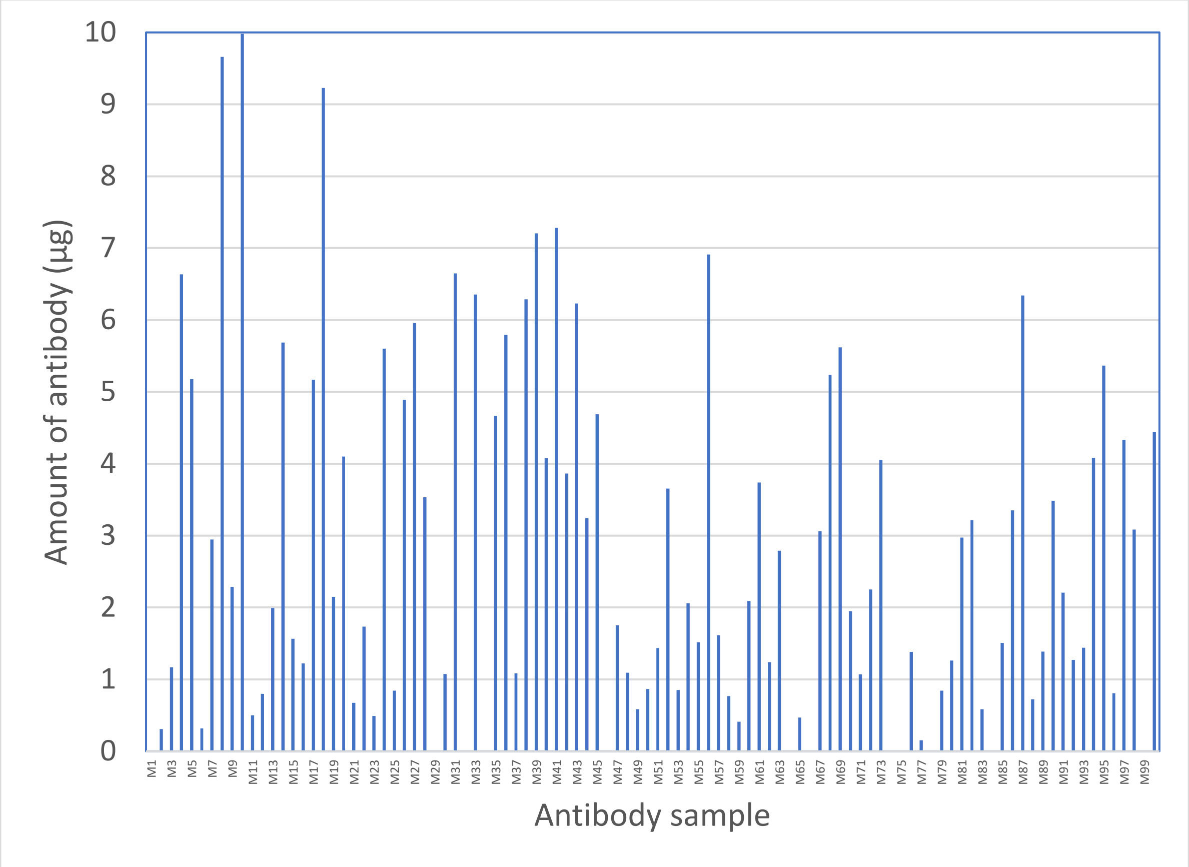

Figure S2. Antibody amount of each sample prior to protein cleavage determined via Bradford assay. For several samples, the concentration of the antibody was below the limit of detection of the Bradford-Assay (~1 µg). In some of these cases, it was not possible to obtain an information-rich fingerprint spectrum. However, the majority of the samples (89 in total), even if below the quantification limit of the Bradford assay, still gave decent fingerprint spectra. Samples with antibody concentrations above 1 µg always resulted in information-rich spectra.

Description of ABID software

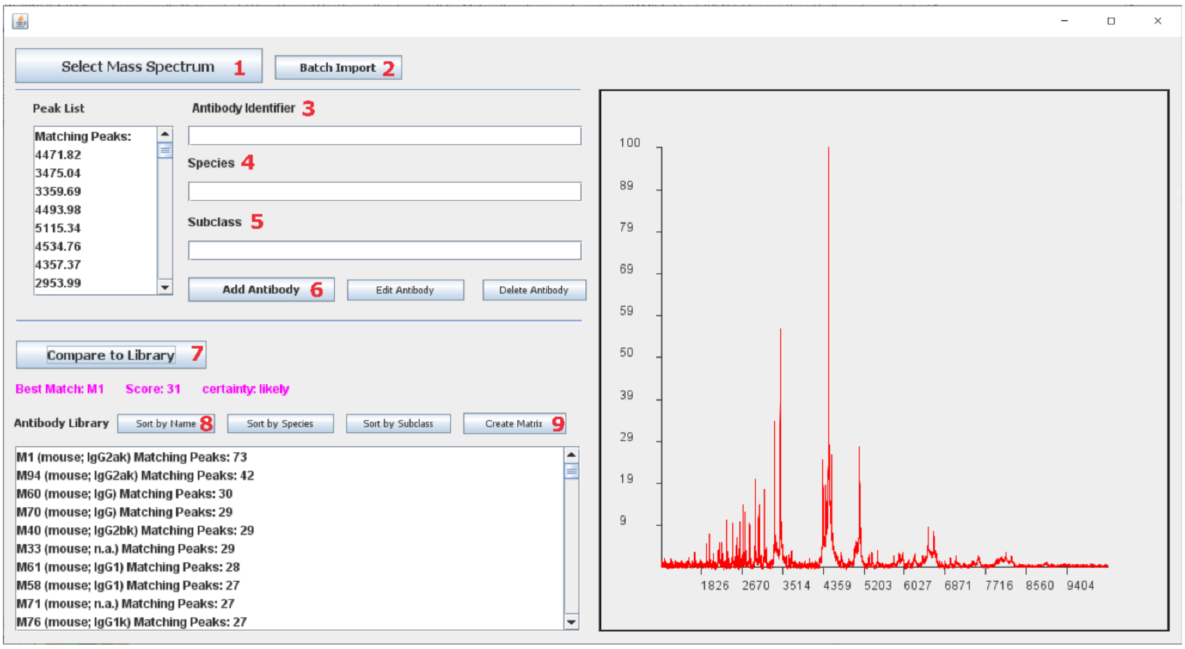

Figure S3. Screenshot of the software tool ABID.

The Software Tool ABID is a standalone Java application running on any device with Java version 8 or higher. Click on ABID.jar to open a new interface showing several buttons and options. Select your ASCII file (e.g., *.txt, *.dat) fingerprint spectrum file ("Select Mass Spectrum") and load it into the software (**1**). For an uploaded spectrum, the name of the antibody must be defined (**3**). If known, the species of origin (**4**) and subclass (**5**) of the antibody can be added. Click on "Add Antibody" (**6**) to add the fingerprint to the library. To compare an uploaded spectrum to a created library, click on "Compare to Library" (**7**). The library is automatically sorted by the number of matching peaks. The user can also sort the library by names of the antibodies, subclass, and species (**8**). To create a correlation matrix, click on "Create Matrix" (**9**). An ASCII file is generated that can easily be imported into Excel.

The attached .zip files to this publication contain the Java software tool ABID and all the 89 antibody fingerprints generated during this study as ASCII files. Using the button "Batch Import" (2), the user can import multiple spectra at once by selecting the directory of the spectra. This way, the same library that the authors used for this work can be easily created and used for further studies.

Specificity of antibody fingerprints (correlation matrix)

Figure S4 shows the correlation matrix for all 89 antibodies used in this study. The number of matching peaks for every antibody is given in a normalized value. The intensity in color correlates with a rising number of matching peaks.

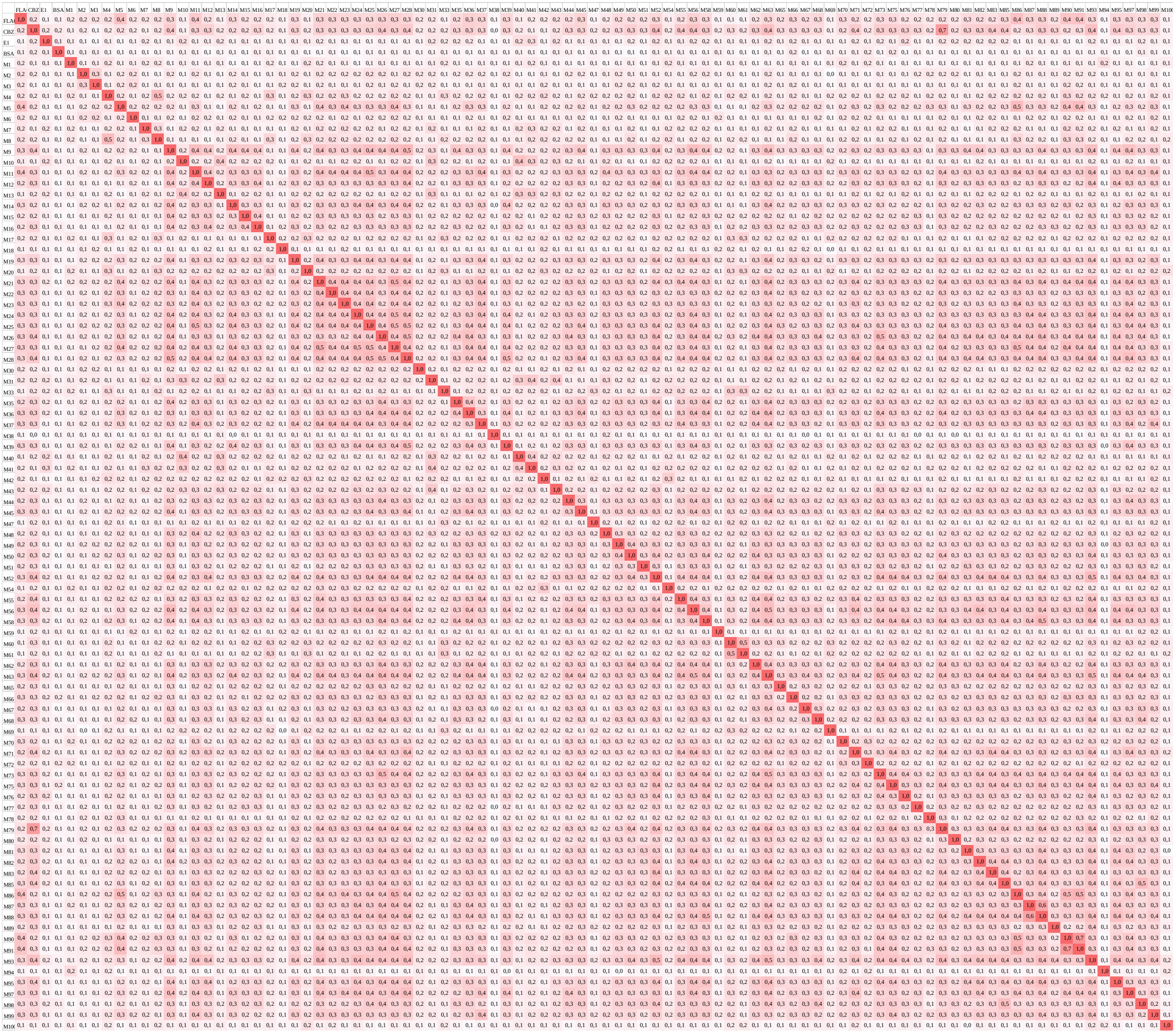

Figure S4. Correlation matrix of all 89 antibodies. The color intensity correlates with an increasing number of matching peaks.

Blinded random sampling

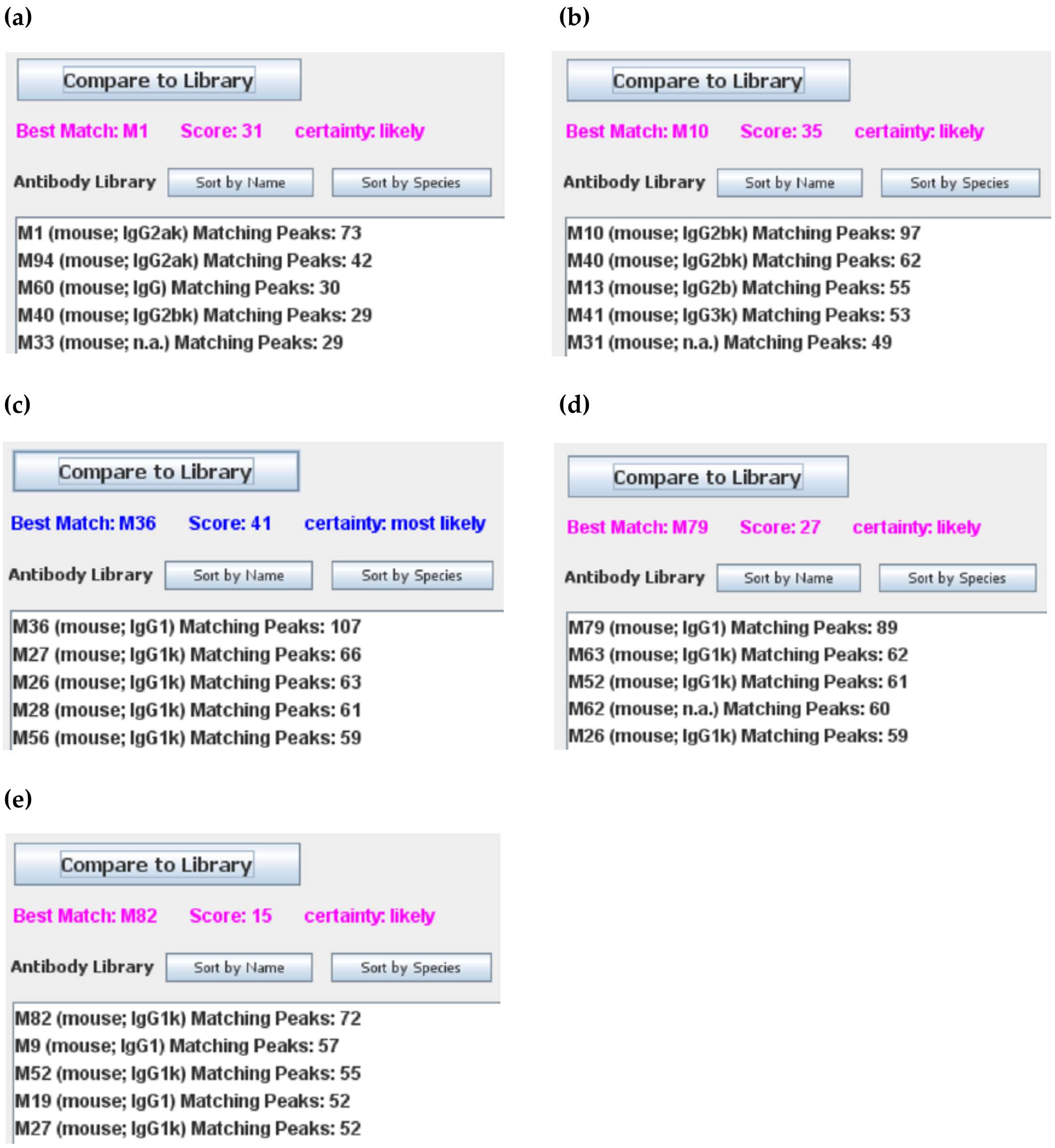

Figure S5. Software-based results of the blinded samples. Randomly selected samples were (a) M1, (b) M10, (c) M36, (d) M79 and (e) M82. Samples were successfully recognized based on the highest number of matching peaks.

Fingerprint spectra of all antibodies

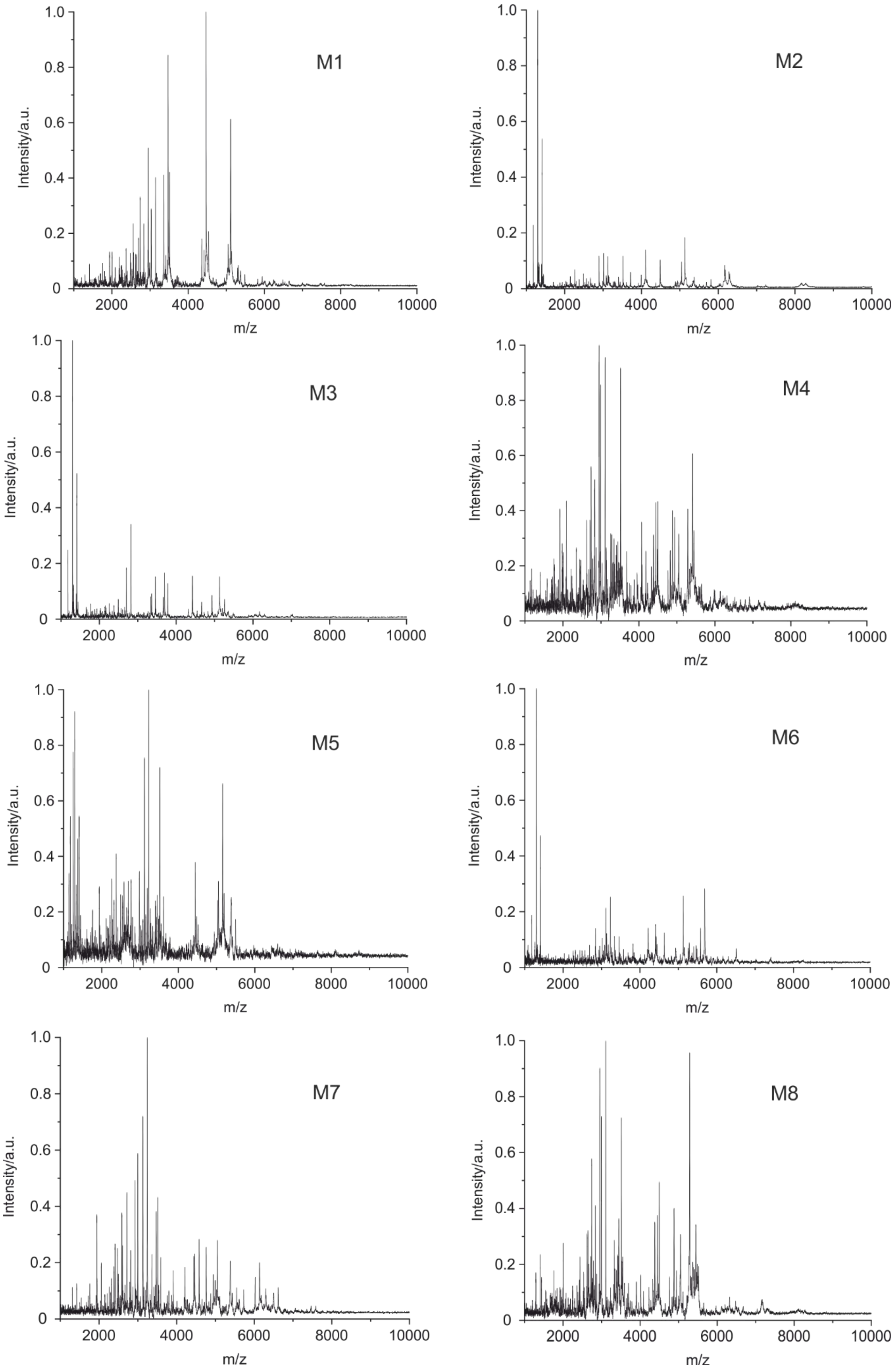

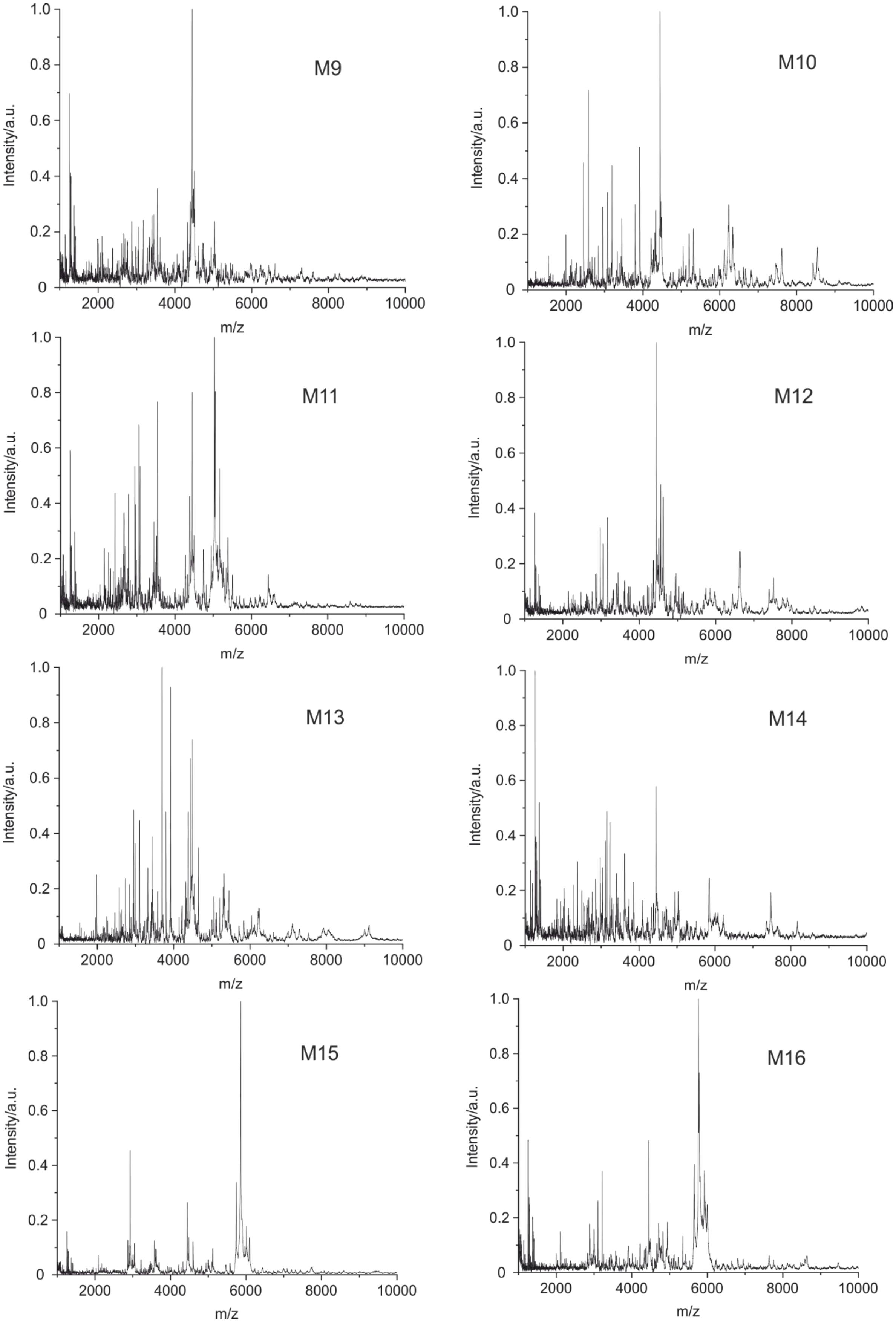
M9
M10
M11
M12
M13
M14
M15
M16
Intensity/a.u.
m/z
1.0
0.8
0.6
0.4
0.2
0
2000
4000
6000
8000
10000

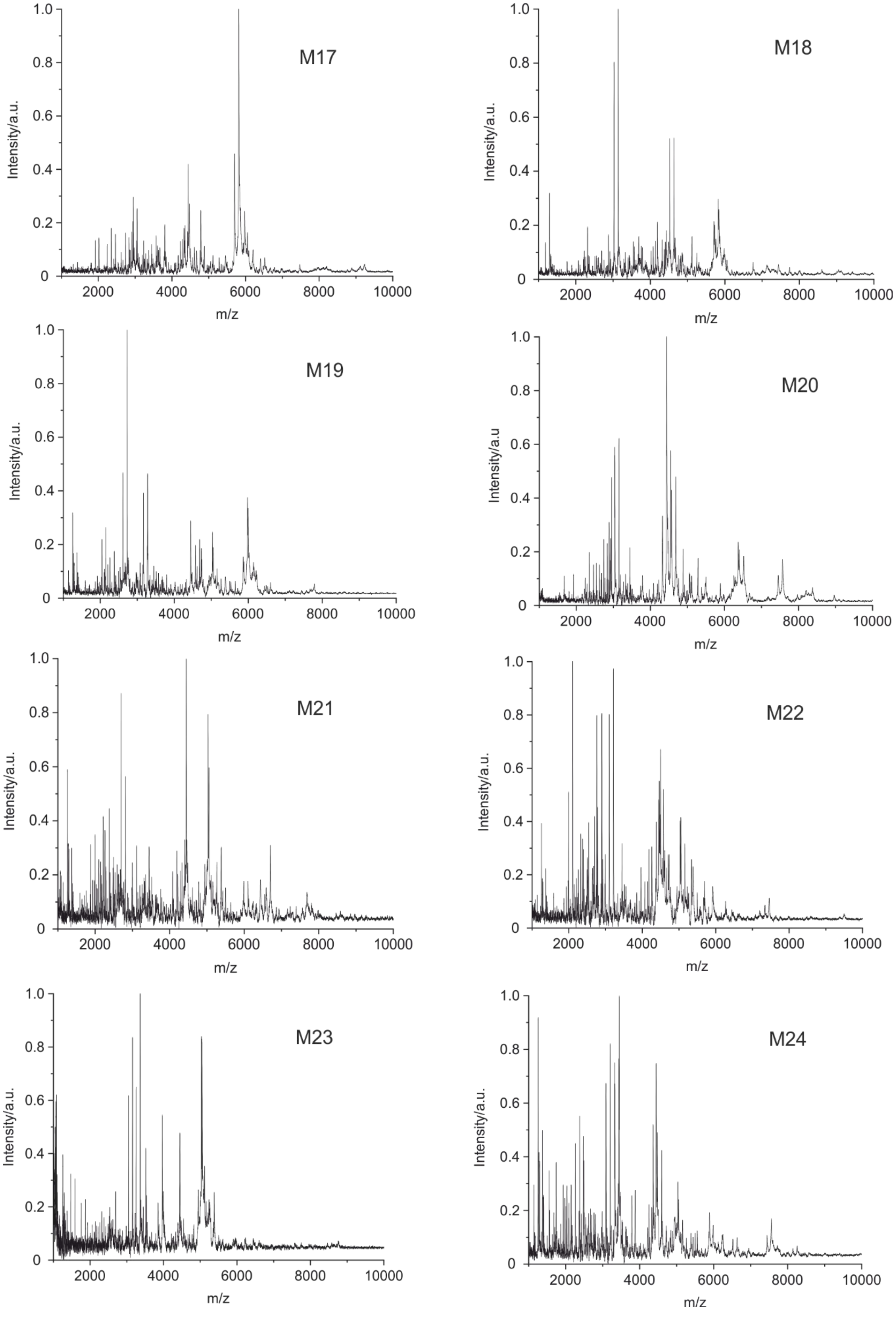
M17
M18
M19
M20
M21
M22
M23
M24
Intensity/a.u.
Intensity/a.u
m/z

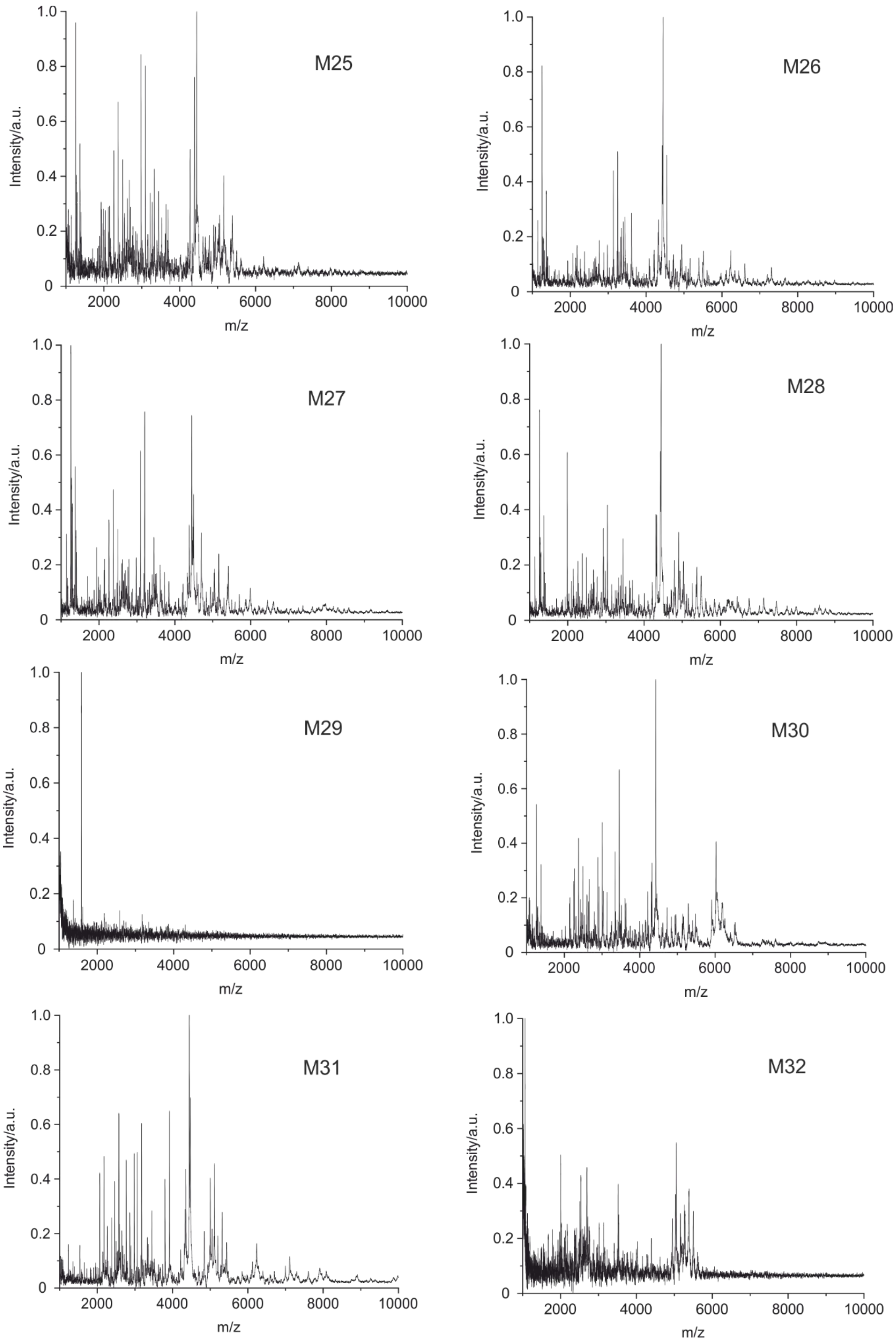
M25
M26
M27
M28
M29
M30
M31
M32
Intensity/a.u.
m/z

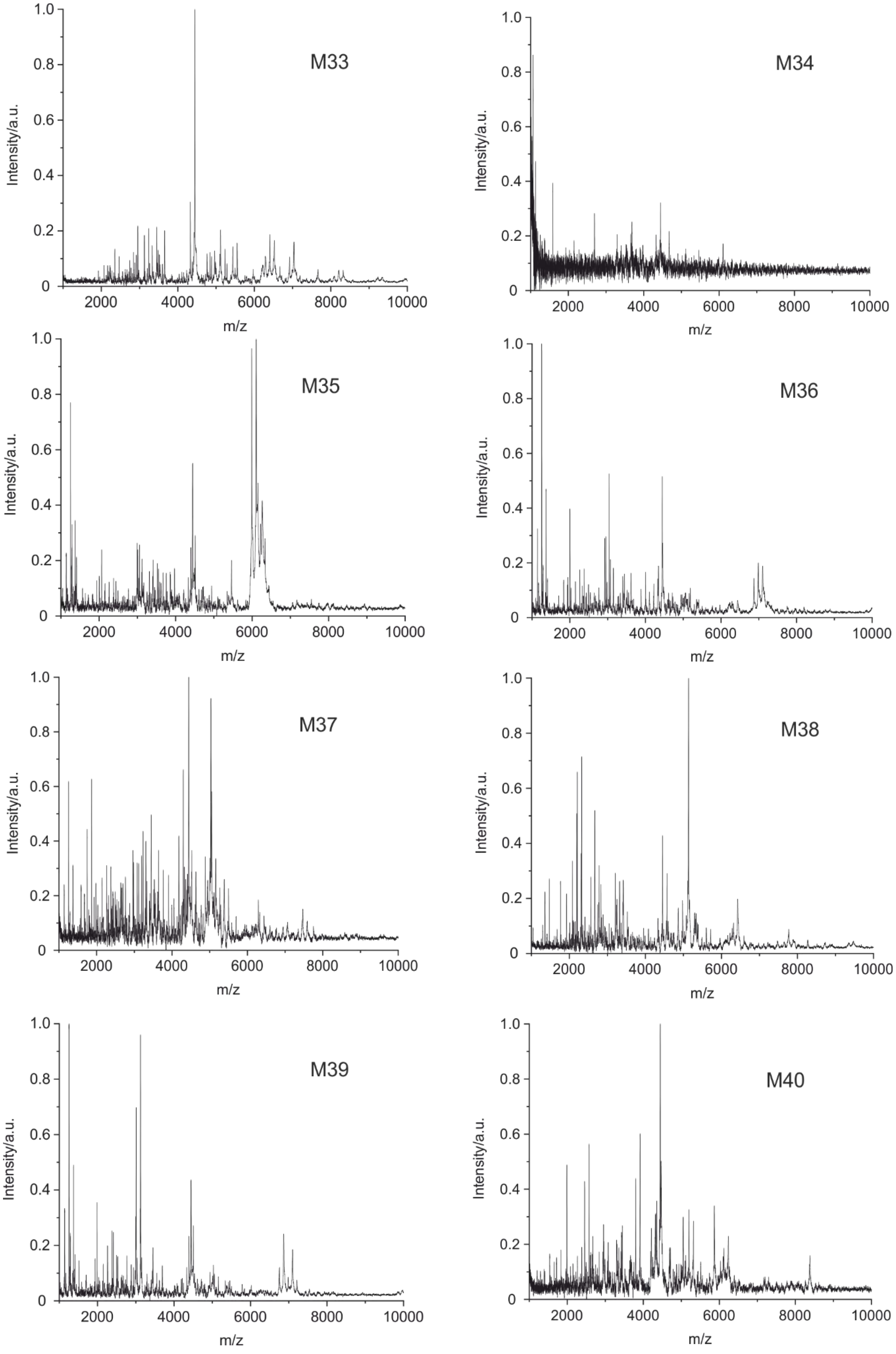
M33
M34
M35
M36
M37
M38
M39
M40
Intensity/a.u.
m/z
0
0.2
0.4
0.6
0.8
1.0
2000
4000
6000
8000
10000

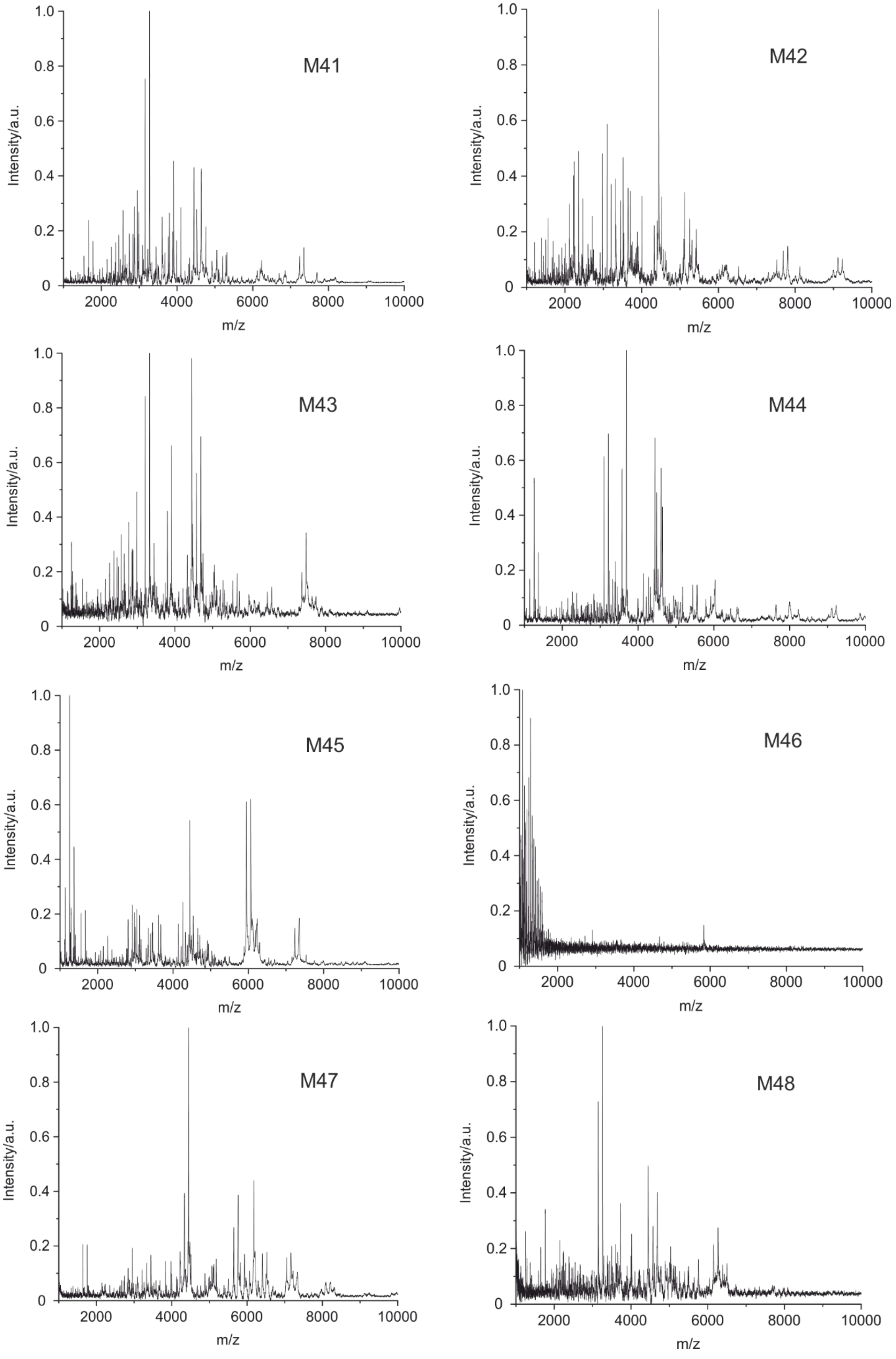
M41
M42
M43
M44
M45
M46
M47
M48
Intensity/a.u.
m/z
0
0.2
0.4
0.6
0.8
1.0
2000
4000
6000
8000
10000

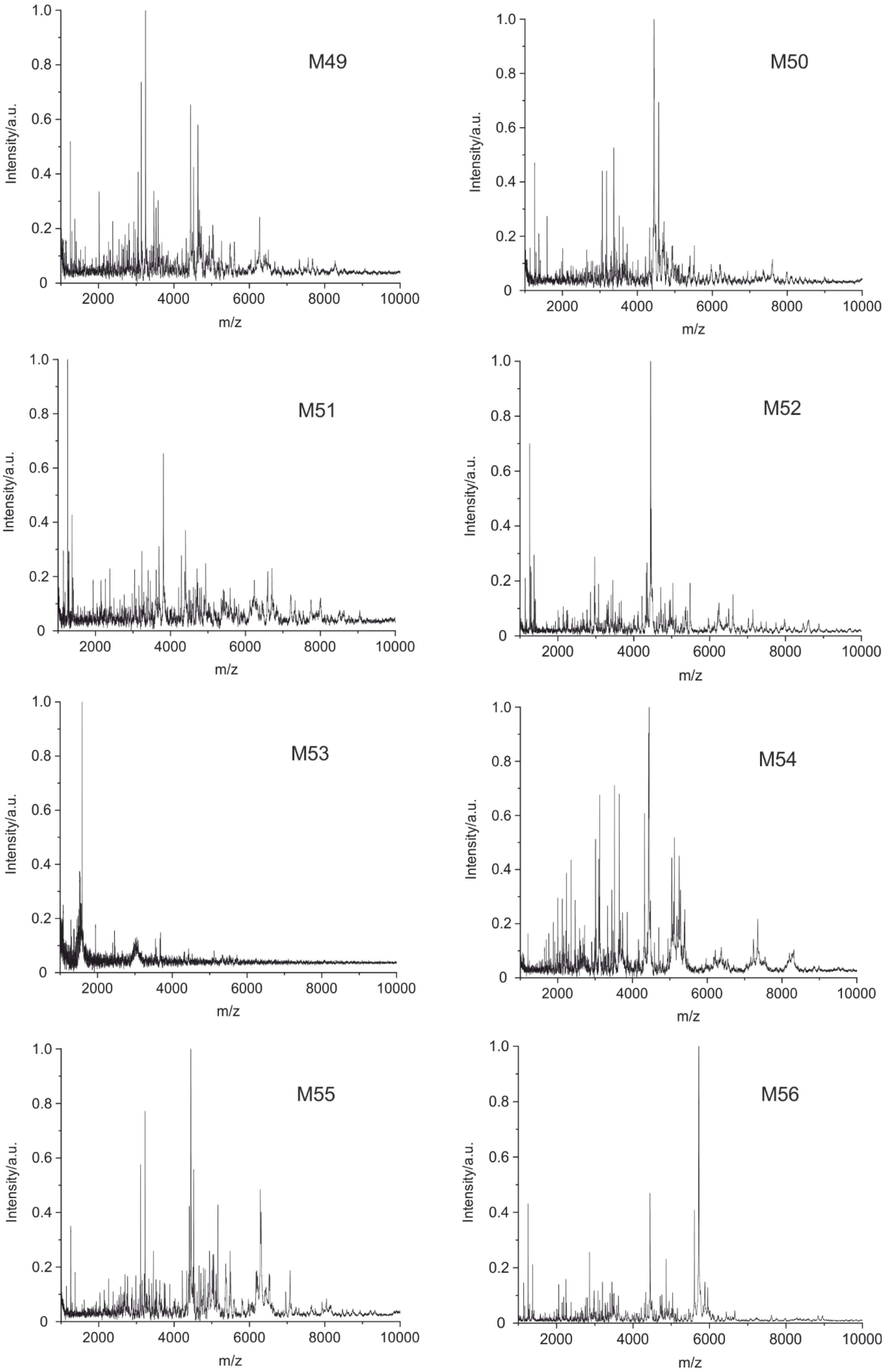
M49
M50
M51
M52
M53
M54
M55
M56
Intensity/a.u.
m/z

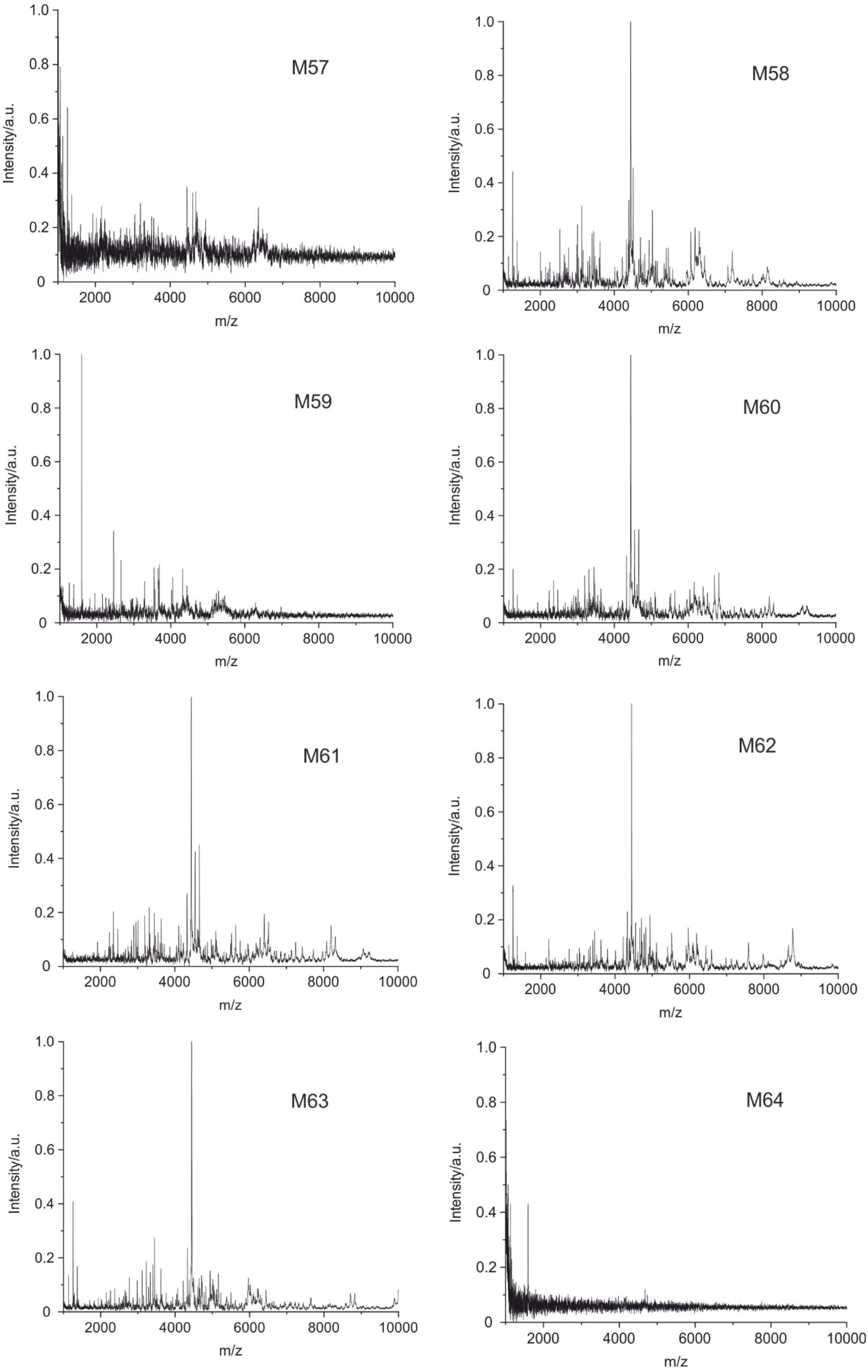
M57
M58
M59
M60
M61
M62
M63
M64
Intensity/a.u.
m/z
0
0.2
0.4
0.6
0.8
1.0
2000
4000
6000
8000
10000

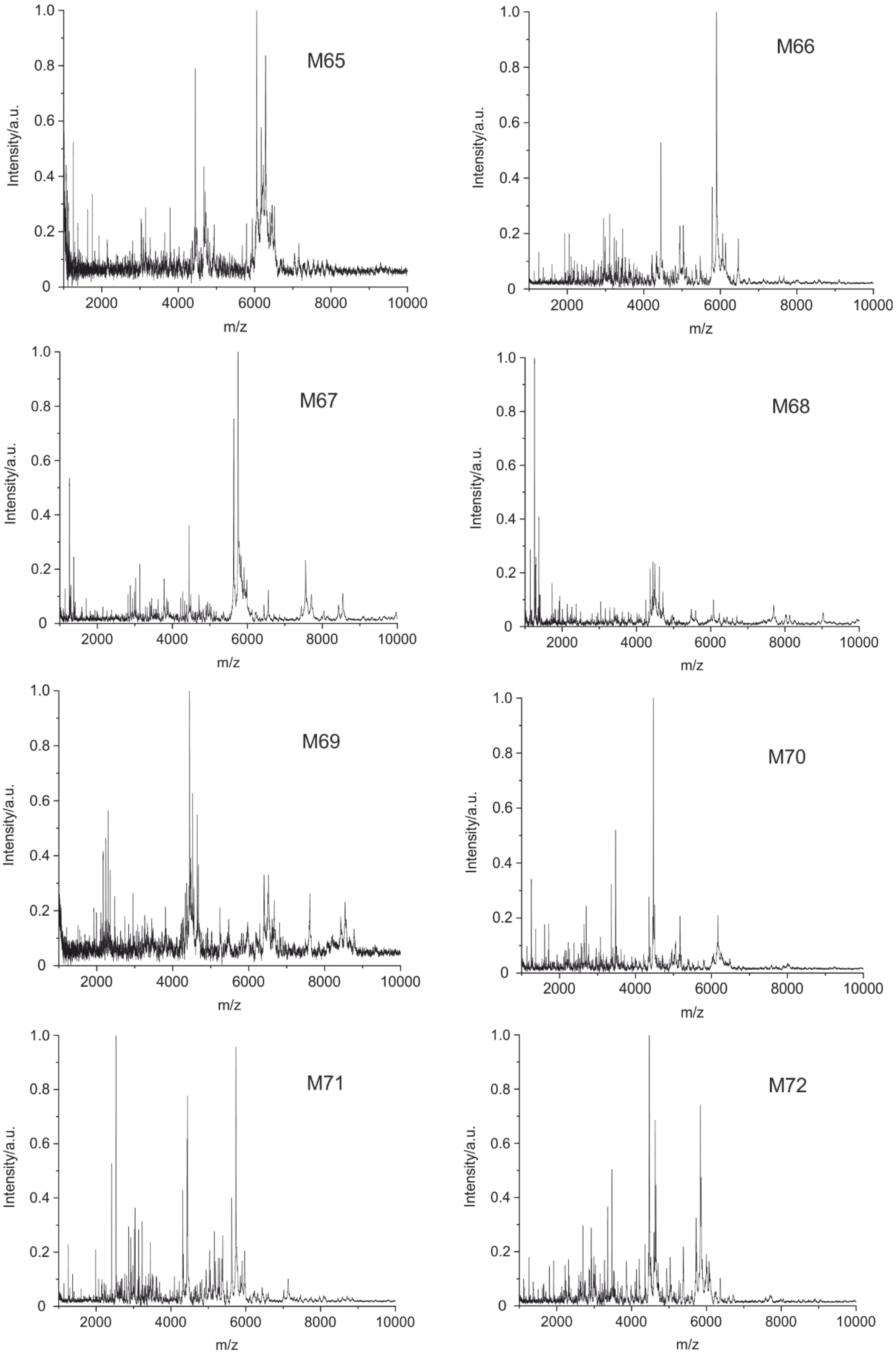
M65
M66
M67
M68
M69
M70
M71
M72
Intensity/a.u.
m/z

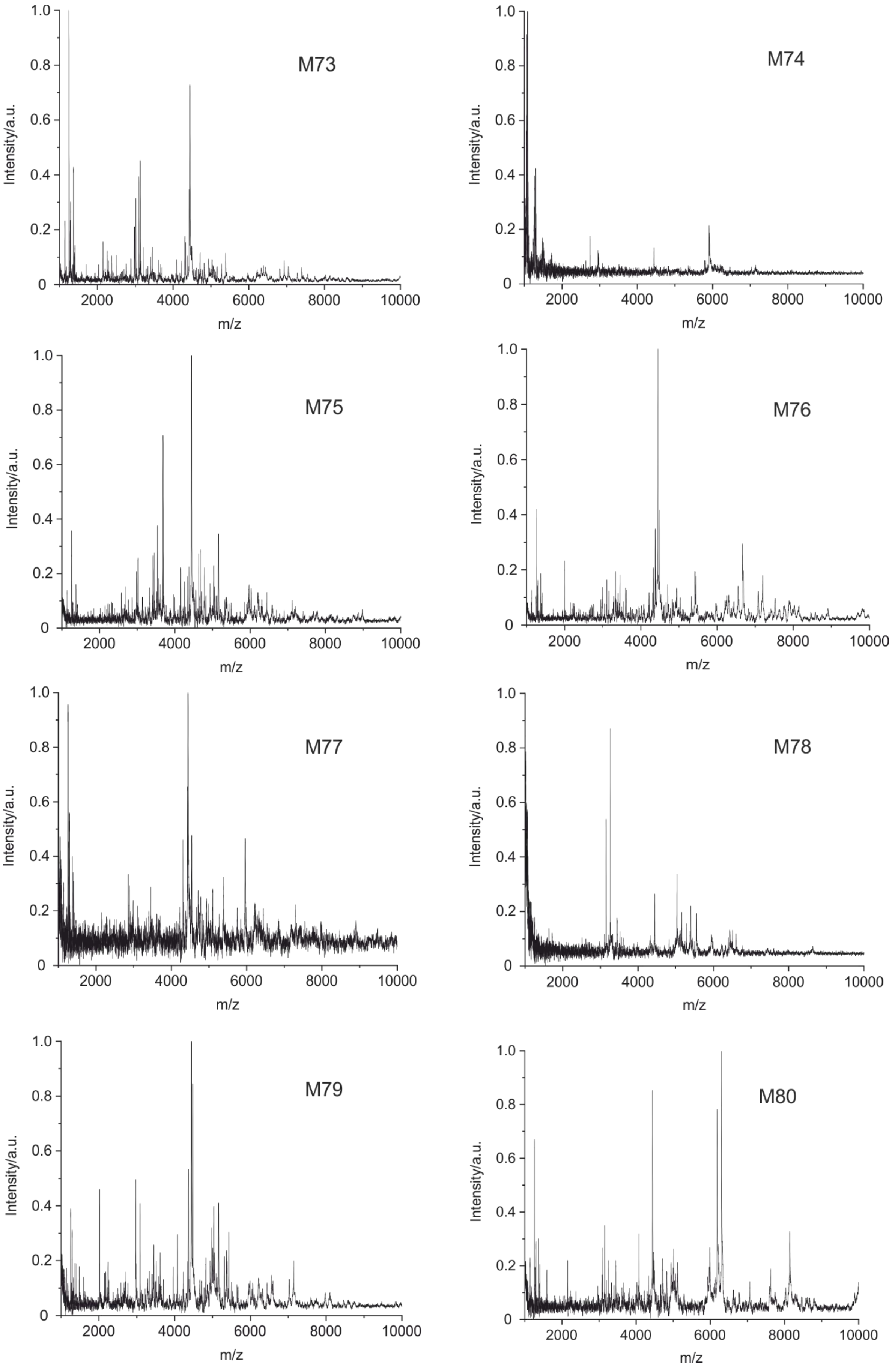
M73
M74
M75
M76
M77
M78
M79
M80
Intensity/a.u.
m/z

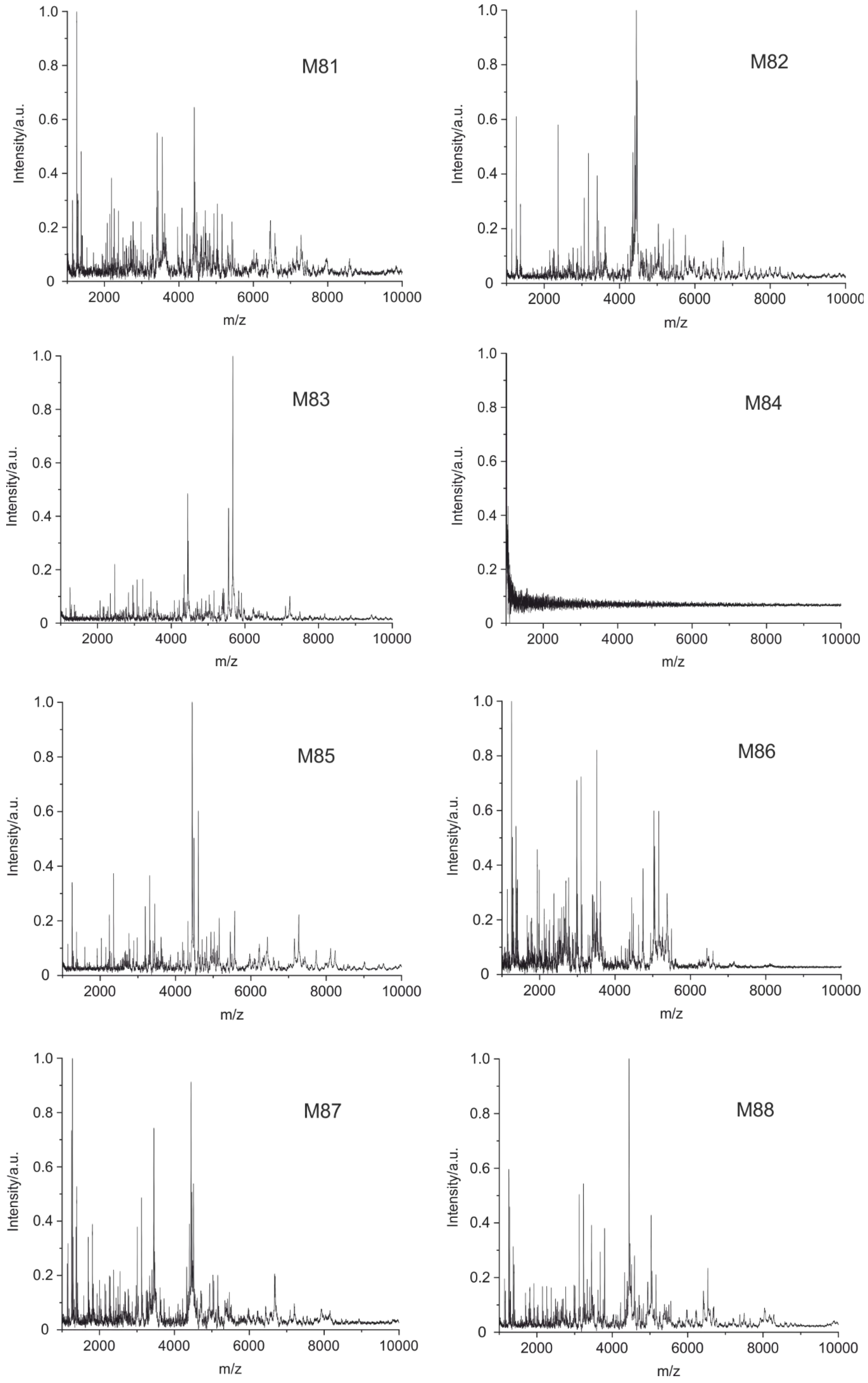
M81
M82
M83
M84
M85
M86
M87
M88
Intensity/a.u.
m/z
0
0.2
0.4
0.6
0.8
1.0
2000
4000
6000
8000
10000

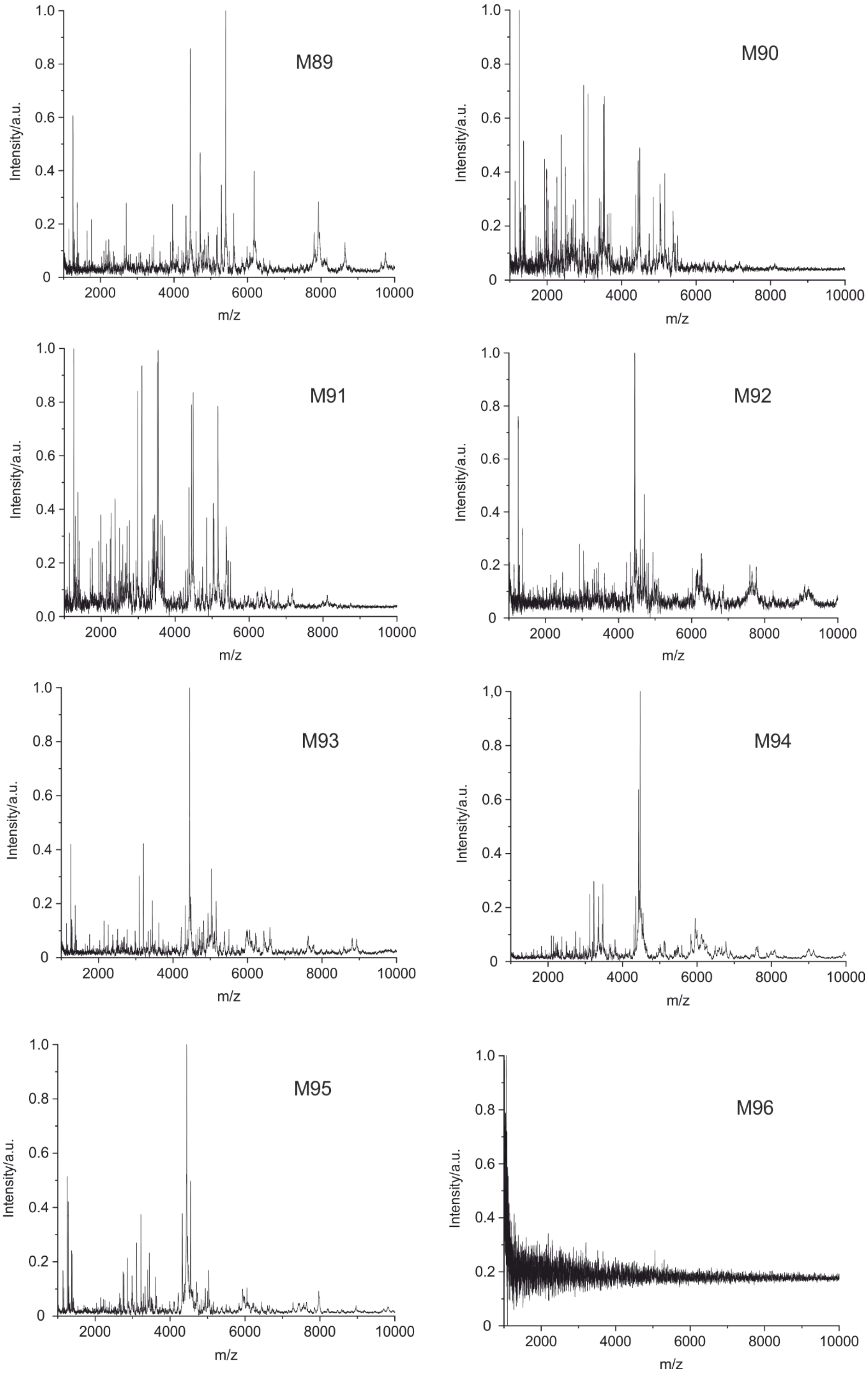
M89
M90
M91
M92
M93
M94
M95
M96
Intensity/a.u.
m/z
0
0.2
0.4
0.6
0.8
1.0
2000
4000
6000
8000
10000

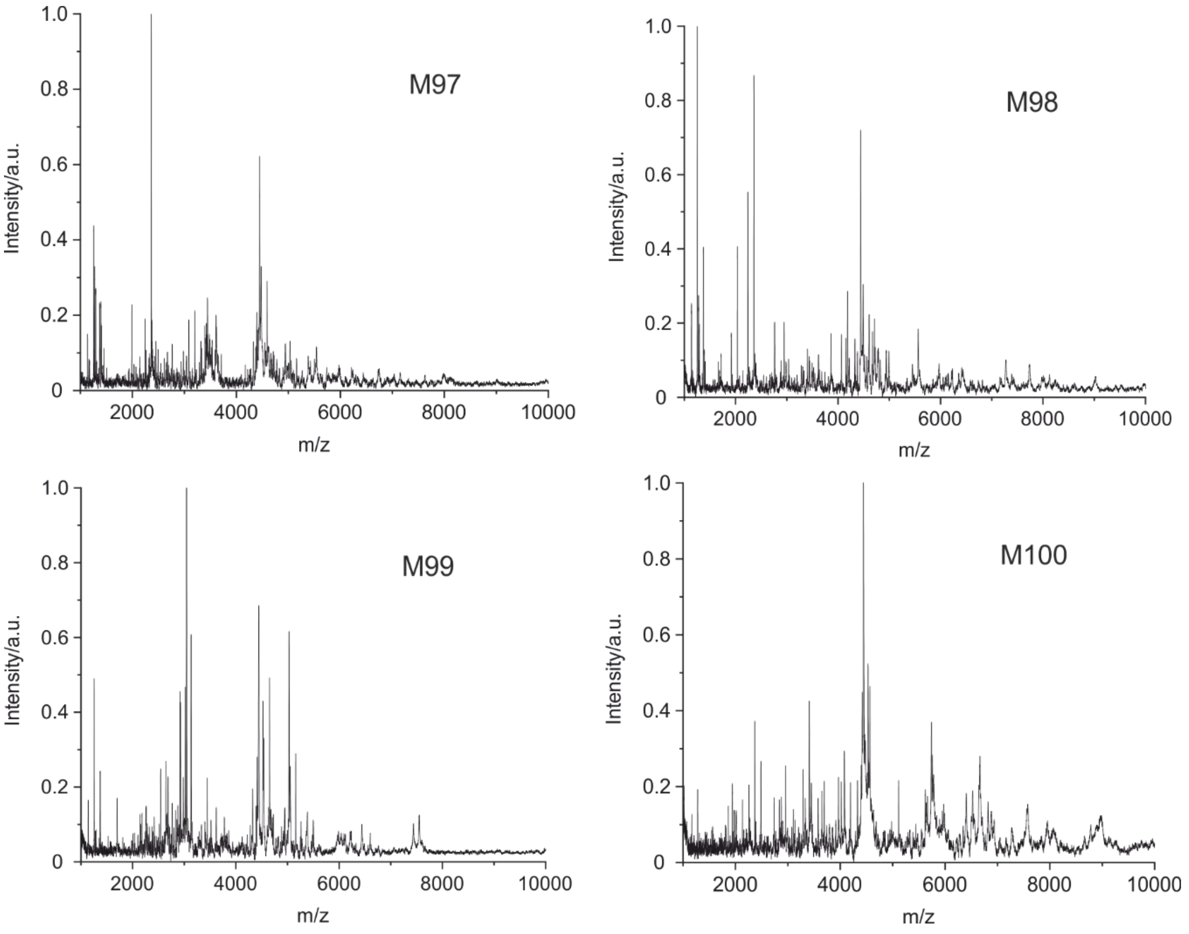
M97
M98
M99
M100
Intensity/a.u.
m/z
1.0
0.8
0.6
0.4
0.2
0
2000
4000
6000
8000
10000
